# Induction of miR 21 impairs the anti-*Leishmania* response through inhibition of IL-12 in canine splenic leukocytes

**DOI:** 10.1371/journal.pone.0226192

**Published:** 2019-12-11

**Authors:** Larissa Martins Melo, Jaqueline Poleto Bragato, Gabriela Lovizutto Venturin, Gabriela Torres Rebech, Sidnei Ferro Costa, Leandro Encarnação Garcia, Flávia Lombardi Lopes, Flávia de Rezende Eugênio, Paulo Sérgio Patto dos Santos, Valéria Marçal Felix de Lima

**Affiliations:** 1 Department of Animal Clinic, Surgery and Reproduction, São Paulo State University (Unesp), School of Veterinary Medicine, Araçatuba,São Paulo, Brazil; 2 Department of Production and Animal Health, São Paulo State University (Unesp), School of Veterinary Medicine, Araçatuba, São Paulo, Brazil; Ohio State University, UNITED STATES

## Abstract

Visceral Leishmaniasis is a chronic zoonosis and, if left untreated, can be fatal. Infected dogs have decreased cellular immunity (Th1) and develop a potent humoral response (Th2), which is not effective for elimination of the protozoan. Immune response can be modulated by microRNAs (miRNAs), however, characterization of miRNAs and their possible regulatory role in the spleen of infected dogs have not been done. We evaluated miRNA expression in splenic leukocytes (SL) from dogs naturally infected with *Leishmania infantum* and developing leishmaniasis (CanL; n = 8) compared to healthy dogs (n = 4). Microarray analysis showed increased expression of miR 21, miR 148a, miR 7 and miR 615, and downregulation of miR 150, miR 125a and miR 125b. Real-time PCR validated the differential expression of miR 21, miR 148a and miR 615. Further, decrease of miR 21 in SL, by means of transfection with a miR 21 inhibitor, increased the IL-12 cytokine and the T-bet/GATA-3 ratio, and decreased parasite load on SL of dogs with CanL. Taken together, these findings suggest that *L*. *infantum* infection alters splenic expression of miRNAs and that miR 21 interferes in the cellular immune response of *L*. *infantum*-infected dogs, placing this miRNA as a possible therapeutic target in CanL.

## Introduction

Visceral Leishmaniasis (VL), caused by the protozoan *Leishmania (L*.*) infantum* [1], is considered one of the most severe forms of the disease [2] and has seen a very significant increase in number of cases in recent years, representing a serious problem to public health [1]. The visceral form of the disease can be found in at least 65 countries, with most cases occurring in Brazil, East Africa and Southeast Asia [3]. It is estimated that 50,000 to 90,000 new cases of VL occur worldwide each year [3]. In humans and dogs, the parasite can cause lesions and symptoms that are characteristic of VL [4,5], with lymphadenopathy, onychogrifosis, cutaneous lesions, weight loss, cachexia and locomotor abnormalities being commonly found in dogs [6].

In CanL, the spleen is one of the most affected organs during infection [7], along with skin and bone marrow [8]. High parasitism is observed in the spleen, leading to significant morphological changes such as hypertrophy and hyperplasia of the red pulp with infiltration of mononuclear cells and mainly plasma cells [9]. Replacement of macrophages by lymphocytes takes place in the white pulp due to hypertrophy and hyperplasia of this area [9]; unlike peripheral blood, the spleen is the place where immune response against the parasite will occur through macrophage and lymphocyte activation. Canine immune response to the parasite is compartmentalized [9], emphasizing the importance of spleen investigations.

In CanL, protective immunity has been associated with a cellular immune response [10], manifested by positive lymphoproliferative response to *Leishmania* spp antigens [11] and cytokine production, such as IFN-γ, TNF-α and IL-12 [10]. These cytokines are required for macrophage activation and death of intracellular parasites [12]. Regulation of the effector function of macrophages and T cells seems to depend on microRNAs (miRNAs), small non-coding RNAs of approximately 21 nucleotides in length that are transcribed in the cell nucleus and function as post-transcriptional regulators of gene expression, controlling translation of key proteins involved in immune response [13]. Recent studies have characterized miRNA profiles in VL [14–17], but the regulatory role of these miRNAs in the immune response is poorly characterized.

In this study, we demonstrate that miRNAs are differentially expressed in SL of dogs with CanL when compared to control dogs. MiR 21 regulation of IL-12 production and polarization of the immune response by Th2, consequently modifying the Th1/Th2 profile, contributes to increased parasite load.

## Materials and methods

### Dog screening and sample collection

This study was approved by the Committee for Ethics in Animal Experimental Research (COBEA), with the approval of the Committee for Ethics in Animal Use (CEUA) of São Paulo State University (UNESP), School of Veterinary Medicine, Araçatuba (process 00978/2016).

Adult dogs of both sexes were selected from the Araçatuba Zoonosis Control Center or from owners who signed a free and informed consent form.

For microarray analysis, four dogs were used as controls. These animals were selected following clinical examination, complete blood count (S1 Table) and serum biochemical profile (S2 Table) within normal range for the species and negative results (serological and molecular diagnosis) for CanL (S3 Table). For the infected group, eight dogs naturally infected by *L*. *infantum* [18], with at least three clinical signs of the disease, including onychogrifosis, weight loss, ear-tip injuries, periocular lesions, alopecia, skin lesions and lymphadenopathy, and with serological and molecular diagnosis positive for leishmaniasis (S3 Table) were chosen for the study. Infected dogs were further classified in the clinical stage II of the disease, based on blood count results (S1 Table) and biochemical profile (S2 Table) [19].

For transfection studies with miScript miRNA Mimic and Inhibitor (Qiagen, USA) in SL, four control dogs with negative serological [20] and molecular diagnosis for leishmaniasis [21] (S4 Table), and eight dogs naturally infected by *L*. *infantum* with positive serological and molecular diagnosis for leishmaniasis (S4 Table) were employed. The same inclusion criteria described above were used for transfection experiments (S5 and S6 Tables).

Blood (5ml) was collected from dogs enrolled in the study by jugular vein puncture, 4ml in tubes without EDTA to obtain serum for indirect ELISA for detection of anti-leishmania antibodies [20] and biochemical profile, and 1ml in tubes with EDTA for complete blood count. Veterinarians at the shelter euthanized the infected dogs using barbiturate anesthesia (Tiopental, Cristália Itapira, SP), followed by intravenous injection of 19.1% potassium chloride by the same route, in compliance with local legislation. Visceral Leishmaniasis Control and Surveillance Program mainly relies on the euthanasia of seropositive dogs to control VL in Brazil.

After euthanasia, a 2cm^3^ fragment of the spleen was collected for isolation of splenic leukocytes. Spleen fragments in control dogs were removed by surgical excision [22].

### Isolation of splenic leukocytes

Total splenic leukocytes were obtained from a 2cm^3^ fragment that was macerated and added to 10ml RPMI-1640 medium (Sigma, USA) supplemented with 10% heat inactivated fetal bovine serum, 0.03% L-glutamine, and 100IU/ml penicillin and 100mg/ml streptomycin. After removal of cell debris through a 100μm filter (BD Falcon Cell strainer, USA), suspension was processed with 5ml of red blood cell lysis buffer containing 7.46g/L ammonium chloride (NH4 ClO3) at 4°C for 10 minutes, centrifuged at 2000rpm for 5 minutes and washed with phosphate buffered saline (PBS) at pH 7.2 three times. Obtained cells were then counted in a Neubauer chamber, and DNA and total RNA were extracted as described below.

### Serological diagnosis by ELISA

Samples were analyzed by ELISA assay using total antigen from lysed promastigotes [20]. The plate was coated with 20μl/ml protein, diluted in coating buffer, pH 9.6.The plates were then incubated overnight at 4°C, then washed three times in PBS containing 0.05% Tween 20 (washing buffer) and saturated for 1 hour with 150 μl/well of a mixture of PBS and 10% FCS at room temperature. Next, the preparation was washed again three times with washing buffer. Blocking buffer/Tween (100μl of serum sample (1/400) diluted in PBS, pH 7.2, containing 0.05% Tween 20 and 10% FCS) was added to each well and incubated at room temperature for 3h, followed by three washes with washing buffer. Subsequently 100μl/well of anti-dog IgG conjugated with horseradish peroxidase (Sigma, St. Louis, MO, USA) at appropriate dilution in blocking buffer/Tween was added, incubated at room temperature for 1 hour and washed. Substrate solution (0.4mg/ml o-phenylenediamine (Sigma, St. Louis, MO, USA) and 0.4μl/ml H2O2 in phosphate citrate buffer, pH 5.0) was added at 100 μl/well and developed for 5 min at room temperature. The reaction was stopped with 50μl of 3 M H2SO4. Absorbance was measured at 490 nm using a Tecan microplate reader (Sunrise model ref. 16039400). Negative and positive controls were included on each plate. Positive controls obtained from a hyperimmune animal were included. The cut-off was determined using the mean + 3 SD of the readings obtained from serum samples of healthy dogs from non-endemic leishmaniasis areas.

### Molecular diagnosis by real-time PCR

Parasite load quantification was performed by Real-Time PCR with a final reaction volume of 20μL using primers amplifying a 116bp fragment from the kinetoplast DNA (kDNA) of *Leishmania* spp. (5 'CCTATTTTACACCAACCCCCAGT 3' and 5 'GGGTAGGGGCGTTCTGCGAAA 3'), at a concentration of 900nM [23], Power SYBR Green PCR Master Mix (Applied Biosystems, USA) and 50ng of DNA. Amplification conditions consisted of an initial denaturing step at 95°C for 10 minutes, followed by 40 cycles of 95°C for 15 seconds and 65°C for 60 seconds. Following amplification, a dissociation curve of the amplified fragment was determined from 60°C to 95°C, with an increase of 0.5°C every 5 seconds. For each reaction, a standard curve with serial dilution of DNA from *Leishmania infantum* promastigotes (MHOM / BR00 / MER02) was performed.

### Extraction and quantification of total RNA

Extraction of total RNA from splenic leukocytes was performed immediately after sample processing, with the commercial mirVana kit for isolation of total RNA with phenol (Life Technologies, USA), following manufacturer’s instructions. After total RNA isolation, sample were stored at -80°C for determination of quality and concentration.

RNA samples were analyzed by spectrophotometer (NanoDrop, Thermo Scientific, USA) for purity evaluation (260/280) and quantified in a fluorimeter (Qubit 3.0, Invitrogen), using Qubit RNA HS Assay Kit (Life Technologies, USA). Before performing microarray, RNA quality of all samples was also evaluated by capillary electrophoresis (Bioanalyzer, Agilent Technologies, USA) using the commercial Agilent RNA 6000 Nano Kit.

### Microarray

Total RNA (250ng) with RNA Integrity Number (RIN) superior to 8 were used to perform microarray analysis using the miRNA 4.1 Array Strip (Affymetrix, USA), containing probes designed for miRNAs of different species.

miRNAs were biotinylated using the Affymetrix FlashTag Biotin HSR RNA Labeling Kit. Hybridization, staining and washes were performed using the GeneAtlas Hybridization, Wash, and Stain Kit for miRNA array Strips. Hybridization was carried out for 20 hours at 48°C, and immediately followed by the GeneAtlas Wash, Stain and Scan protocol.

Microarray data were deposited in the Gene Expression Omnibus database with accession number GSE112459 (https://www.ncbi.nlm.nih.gov/geo/query/acc.cgi?acc=GSE112459) according to the Minimum Information About Microarray Experiment (MIAME) standards.

### Microarray data analysis

Normalization and quality control of microarray results for control and infected dogs were performed in the Expression Console Software, version 1.4.1 (Affymetrix, Thermo Fisher Scientific, USA). Differential expression analysis of miRNAs was performed in the Transcriptome Analysis Console software (Affymetrix, Thermo Fisher Scientific, USA).

Targets of differentially expressed miRNAs in dogs with CanL, and their canonical pathways were analyzed using the Ingenuity Pathway Analysis program (Qiagen, USA). Enrichment analysis of Gene Ontology (GO) terms was performed using the ENRICHR program (http://amp.pharm.mssm.edu/Enrichr/) [24,25].

### Real-time PCR for miRNAs validation

Microarray results were validated by real-time PCR (qPCR). miScript II RT kit (Qiagen) was used for cDNA production from total RNA samples. A total of 1μg of RNA was used for each sample with the 5x miScript Hiflex Buffer, in a final volume of 20μl. Mix was incubated for 60 min at 37°C, followed by 5 min at 95°C to inactivate the miScript Reverse Transcriptase. Next, qPCR was performed using commercially available specific primers for the *Canis familiaris* miRNAs and the endogenous reference SNORD96A (miScript, Qiagen). SYBR Green system (myScript SYBR Green PCR kit, Qiagen) was used in a real-time thermal cycler (RealPlex, Eppendorf). Amplification conditions consisted of an initial activation step of 95°C for 15 min followed by 40 cycles of 94°C for 15 seconds, 55°C for 30 seconds and 70°C for 30 seconds, for denaturation, annealing and extension, respectively. For each miRNA, a standard curve was performed with serial dilution of a pool of cDNAs. Absolute quantification of each miRNA was performed by converting the sample cycle threshold values to a concentration (ng/μl), based on the standard curves, which were generated using 10-fold serial dilutions of the cDNA pool. Values obtained for each target miRNA were then divided by SNORD96A values, in order to obtain normalized target values for each sample. All samples were run in duplicate.

### Transfection to increase or decrease miR 21 in splenic leukocytes

Splenic leukocytes were cultured (10^4^ cells/replicate) in triplicate in a 96-well plate for 67h at 37°C in 5% CO2. All Stars Negative control siRNA (scrambled), miR 21 mimic, miR 21 inhibitor (miScript miRNA Mimic and Inhibitor Qiagen, USA) were used at a final concentration of 75nM and were transfected using 1.5μL of Hiperfect (Qiagen, USA) in each well. For the evaluation of transfection rates, AllStars HS Cell Death Control siRNA (Qiagen, USA) was used at a final concentration of 75nM. A transfection rate of >40%, as measured by trypan blue and analyzed by optical microscopy, was set as a cut off value.

### Dosage of IL-12

After transfection, SL were incubated for 67h at 37°C in the 5% CO_2_ and supernatants were collected, centrifuged at 2500rpm and stored at -80°C until further analysis. Concentration of IL-12 in the supernatant was determined by capture ELISA using Canine IL-12/IL-23 p40 DuoSet ELISA Kit (R&D Systems, USA). Initially, plate was prepared with capture antibody and incubated overnight at room temperature. Then, washed three times with Wash Buffer. Plate was blocked by adding 300μl of Reagent diluent to each well, then incubated for 1h at room temperature and washed three times with Wash Buffer. A volume of 100μl of sample or standards in Reagent Diluent was added per well, incubated for 2h at room temperature and washed three times with Wash Buffer. Next, 100μl of Streptavidin-HRP was added to each well, incubated for 20 minutes at room temperature and washed three times with Wash Buffer. Revelation was performed with 100μl of substrate solution per well, followed by a 20-minute incubation at room temperature. Lastly 50μl of Stop Solution were added to each well. Results were expressed in pg/ml. The lower limit of detection of the standard curve was 3.5 pg/ml.

### Quantification of parasite load in cell culture by flow cytometry

After transfection, SL were incubated for 67 h at 37°C in the 5% CO_2_, and parasite load was determined by flow cytometry. The method described by [26] was used, with modifications. SL (10^4^ cells / treatment) were fixed with 1% paraformaldehyde for 60 min at room temperature and permeabilized in ethanol for 60 min at -20°C. SL were incubated with *Leishmania* gp63 monoclonal antibody mouse IgG2a non-conjugated (ABD, Serotec, USA) at 4°C for 60 min. After three successive washes with PBS at pH 7.2 with 2% BSA (bovine serum albumin), cells were stained with secondary antibody goat anti-mouse IgG2a conjugated to phycoerythrin (PE) (R&D Systems, USA), and monoclonal antibody anti-human CD14 conjugated to fluorescein isothiocyanate (FITC) (Bio Rad, USA) for 60 minutes at 4°C. After incubation with secondary antibody, SLs were washed in PBS at pH 7.2 with 2% BSA, and resuspended in PBS at pH 7.2. Flow cytometry was performed on an Accuri C5 cytometer (BD Biosciences, USA). Acquisition of 10,000 events was counted for each replicate on channel FL1 and FL2, and data analysis was performed using the BD Accuri C6 software (version 1.0.264; BD Bioscience, CA, USA). Cells were gated on monocytes (CD14+ cells) and positivity for gp63+ was considered in the analysis.

### Flow cytometry analysis of T-bet (Th1) and GATA 3 (Th2) transcription factors in splenic leukocytes

For T-bet and GATA-3 staining, SL were fixed with 500μl of fixation buffer (R&D systems, USA) and incubated for 10 min at room temperature. Cells were centrifuged at 2000rpm for 5 minutes and washed twice with PBS. Then, SL were resuspended with 150 μl of permeabilization buffer (R&D systems, USA). Cells were incubated with FITC conjugated anti-human monoclonal antibody T-bet (R&D Systems, USA) and with PE conjugated anti-human monoclonal GATA3 (R&D Systems, USA), and control isotypes (R&D Systems, USA). According to a previous study that assessed the similarities between T-bet and GATA-3 protein sequences of *Homo sapiens* and *Canis lupus familiaris* using BLAST (basic local alignment search tool) algorithm of the National Center for Biotechnology Information (NCBI), human T-bet and GATA-3 showed, respectively, 93% and 97% homology with the canine protein. Acquisition of 10,000 events were counted by experimental replicate on channel FL1 and FL2, and cytometric analysis was performed with an Accuri C5 Flow Cytometer (BD Biosciences, USA) using BD Accuri C6 software, version 1.0.264.21 (BD Biosciences, CA, USA).

Lymphocytes were gated by forward and side scatter. Mean fluorescence obtained on T-bet positive cells was divided by the mean fluorescence of positive GATA-3 cells, generating a T-bet (Th1)/GATA-3 (Th2) ratio for each treatment.

### Statistical analysis

Statistical analysis were performed using the GraphPad Prism 6 software (GraphPad Software, Inc., La Jolla, CA, USA). For the microarray, analysis of variance (ANOVA) was used for group comparison. Real-time PCR values for miRNAs were tested by Mann-Whitney test. T-bet, GATA-3, IL-12 dosage and parasite load were evaluated by Friedman Test with multiple comparison. Differences were considered significant when p<0.05.

## Results

### Differentially expressed miRNAs in SL of dogs with CanL

**Fig 1:**
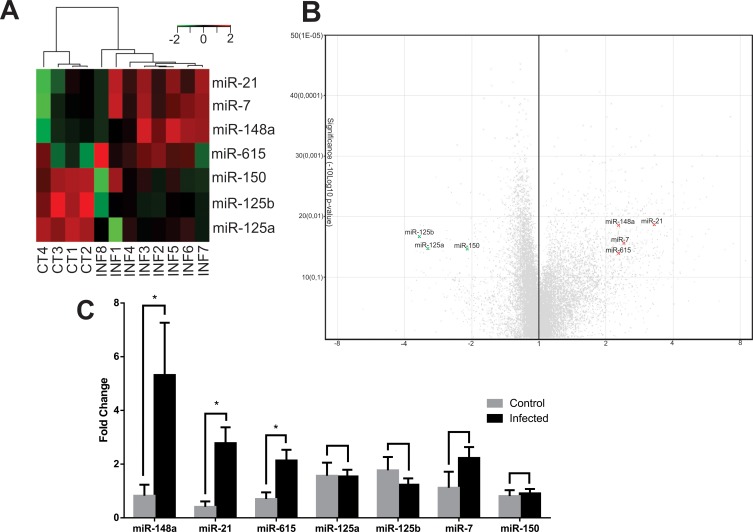
Differentially expressed miRNAs in SL of dogs with CanL. (A) Representation of the 7 miRNAs differentially expressed in SL of dogs with CanL in comparison to healthy dogs. Heat map lines represent individual miRNAs and the columns represent individual samples (eight dogs with CanL and four control dogs). Color scale represents normalized expression levels of miRNAs in the two conditions on the log2 scale; red denotes up-regulation and green denotes down-regulation. (B) Volcano plot. On the left are miRNAs with negative expression (green) and miRNAs with positive expression (red). Analysis of variances (ANOVA) was used between groups, and significance was considered at Fold Change greater than +/-2 and p<0.05. (C) Expression of miRNAs in the infected and control groups was quantified by real-time PCR in SL. Data represent mean of miRNA expression + standard error of the mean. Asterisks represent significance (p<0.05) by Mann-Whitney test.

Given that immunological responses can be regulated by miRNAs [18], we performed microarray for a comparative analysis of miRNA expression in SL of dogs with CanL and control dogs. We observed that miR 21, miR 148a, miR 7 and miR 615 showed increased expression (3.2, 2.3, 2.4 and 2.3 times, respectively), while a decrease in miR 150, miR 125a, miR 125b was observed in CanL (Fig 1A and 1B). To confirm the results, real-time PCR validation was performed. Increase in miR 148a, miR 615 and miR 21 in the SL of the infected group was confirmed by qPCR, similarly to microarray results, as shown in Fig 1C.

### Target genes and their canonical pathways regulated by differentially expressed miRNAs in the spleen of infected animals

With the aid of the IPA target filter tool (highly predicted and experimentally observed targets only), we observed that 114 canonical pathways were regulated by the differentially expressed miRNAs and gene targets (S7 Table). Top 30 canonical pathways and target genes are shown in Table 1, including p53 signaling, PTEN signaling, STAT3 pathway, death receptor and crosstalk signaling between dendritic cells, p38 MAPK signaling, and activation of Th1/Th2 pathways, which are known to regulate immune response in CanL.

**Table 1:** Thirty major canonical pathways predicted for differentially expressed miRNAs in CanL.

Ingenuity Canonical Pathways	P value	Target miRNAs	Target genes
Neuregulin Signaling	0.0001	miR 148a	CDK5R1, ERBB3, ERRFI1, MRAS, NRAS, PRKCZ, PTEN, SOS1, SOS2, TGFA
		miR 21	PIK3R1, PTEN
		miR 615	PRKCG
STAT3 Pathway	0.0010	miR 148a	FLT1, MAP3K9, MRAS, NRAS, TGFA
		miR 21	BMPR2, CDC25A, CDKN1A, SOCS5, TGFBR2
		miR 615	MAPK13
PTEN Signaling	0.0010	miR 148a	FLT1, MRAS, NRAS, PRKCZ, PTEN, SOS1, SOS2
		miR 21	BMPR2, CDKN1A, FASLG, PIK3R1, PTEN, TGFBR2
HER-2 Signaling in Breast Cancer	0.0015	miR 148a	ERBB3, MRAS, NRAS, PRKCZ, SOS1, SOS2
		miR 21	CDK6, CDKN1A, PIK3R1
		miR 615	PRKCG
Myc Mediated Apoptosis Signaling	0.0015	miR 148a	MRAS, NRAS, PRKCZ, SOS1, SOS2
		miR 21	APAF1, FAS, FASLG, PIK3R1
Glioma Signaling	0.0018	miR 148a	MRAS, NRAS, PRKCZ, PTEN, SOS1, SOS2, TGFA
		miR 21	CDK6, CDKN1A, PIK3R1, PTEN
		miR 615	PRKCG
Cytotoxic T Lymphocyte-mediated Apoptosis of Target Cells	0.0019	miR 148a	HLA-A, HLA-B, HLA-C
		miR 21	APAF1, FAS, FASLG
Neurotrophin/TRK Signaling	0.0019	miR 148a	MRAS, NRAS, SOS1, SOS2
		miR 21	NTF3, PIK3R1, SPRY1, SPRY2
		miR 615	NTF4
ErbB Signaling	0.0019	miR 148a	ERBB3, MRAS, NRAS, PRKCZ, SOS1, SOS2, TGFA
		miR 21	PIK3R1, MAPK13
		miR 615	PRKCG
Cholecystokinin/Gastrin-mediated Signaling	0.0021	miR 148a	CCKBR, FLT1, MRAS, NRAS, PRKCZ, ROCK1, SOS1, SOS2
		miR 21	ACTA2, EIF1AX, PIK3R1, TNF
		miR 615	MEF2A, PRKCG
Type I Diabetes Mellitus Signaling	0.0026	miR 148a	HLA-A, HLA-B, HLA-C
		miR 21	APAF1, FAS, FASLG, IL12A, SOCS5, TNF
		miR 615	MAPK13
TGF-β Signaling	0.0032	miR 148a	INHBB, MRAS, NRAS, SOS1, SOS2
		miR 21	BMPR2, SMAD7, TGFBR2
		miR 615	MAPK13
VEGF Family Ligand-Receptor Interactions	0.0032	miR 148a	FLT1, MRAS, NRAS, NRP1, PRKCZ, SOS1, SOS2
		miR 21	PIK3R1
		miR 615	PRKCG
Molecular Mechanisms of Cancer	0.0038	miR 148a	CDK19, MRAS, NRAS, PRKCZ, SOS1, SOS2
		miR 21	APAF1, BMPR2, CDC25A, CDK6, CDKN1A, FAS, FASLG, PIK3R1, SMAD7, TGFBR2
		miR 615	MAPK13, MAX, PRKCG, RALBP1
Th2 Pathway	0.0056	miR 148a	BHLHE41, HLA-A, HLA-B, HLA-DQB2, MAF, S1PR1
		miR 21	BMPR2, IL12A, JAG1, PIK3R1, TGFBR2
VEGF Signaling	0.0071	miR 148a	FLT1, MRAS, NRAS, ROCK1, SOS1, SOS2
		miR 21	ACTA2, EIF1AX, PIK3R1
Th1 and Th2 Activation Pathway	0.0071	miR 148a	BHLHE41, HLA-A, HLA-B, HLA-DQB2, MAF, S1PR1
		miR 21	BMPR2, IL12A, IL6R, JAG1, PIK3R1, TGFBR2
Graft-versus-Host Disease Signaling	0.0071	miR 148a	HLA-A, HLA-B, HLA-C
		miR 21	FAS, FASLG, TNF
p53 Signaling	0.0071	miR 148a	GADD45A, JMY, MDM4, PTEN
		miR 21	APAF1, CDKN1A, FAS, PIK3R1, PTEN, SERPINB5
Prolactin Signaling	0.0071	miR 148a	MRAS, NRAS, PRKCZ, SOS1, SOS2
		miR 21	PIK3R1, SOCS5
		miR 615	PRKCG
Crosstalk between Dendritic Cells and Natural Killer Cells	0.0071	miR 148a	HLA-A, HLA-B, HLA-C
		miR 21	ACTA2, FAS, FASLG, IL12A, TNF
JAK/Stat Signaling	0.0071	miR 148a	CCKBR, MRAS, NRAS, SOS1, SOS2
		miR 21	CDKN1A, PIK3R1, SOCS5
Cell Cycle: G2/M DNA Damage Checkpoint Regulation	0.0077	miR 148a	GADD45A, MDM4, PRKCZ, SKP1
		miR 21	CDKN1A, SKP2
Virus Entry via Endocytic Pathways	0.0085	miR 148a	HLA-A, HLA-B, HLA-C, MRAS, NRAS, PRKCZ
		miR 21	ACTA2, PIK3R1
		miR 615	PRKCG
HGF Signaling	0.0100	miR 148a	MAP3K9, MRAS, NRAS, PRKCZ, SOS1, SOS2
		miR 21	CDKN1A, PIK3R1
		miR 615	PRKCG
p38 MAPK Signaling	0.0102	miR 148a	RPS6KA5
		miR 21	FAS, FASLG, TGFBR2, TNF
		miR 615	HSPB7, MAPK13, MAX, MEF2A
ErbB2-ErbB3 Signaling	0.0104	miR 148a	ERBB3, MRAS, NRAS, PTEN, SOS1, SOS2
		miR 21	PIK3R1, PTEN
Antigen Presentation Pathway	0.0112	miR148a	HLA-A, HLA-B, HLA-C, HLA-DQB2, PDIA3
Fc Epsilon RI Signaling	0.0112	miR 148a	MRAS, NRAS, PRKCZ, SOS1, SOS2
		miR 21	PIK3R1, TNF
		miR 615	MAPK13, PRKCG
NGF Signaling	0.0112	miR 148a	MAP3K9, MRAS, NRAS, PRKCZ, ROCK1, RPS6KA5, SOS1, SOS2
		miR 21	PIK3R1

To understand the functional networks of the target genes, Gene Ontology (GO terms) were analyzed. Analysis showed that these target genes were involved in a large number of biological processes where from a total of 1,101 entries, 30 are presented in S8A Table; for cellular component, of a total of 74 entries, 30 are presented in S8B Table; and for molecular function, a total of 151 entries were found, and 30 are presented in S8C Table.

### miR 21 plays an important role in the inhibition of IL-12

**Fig 2:**
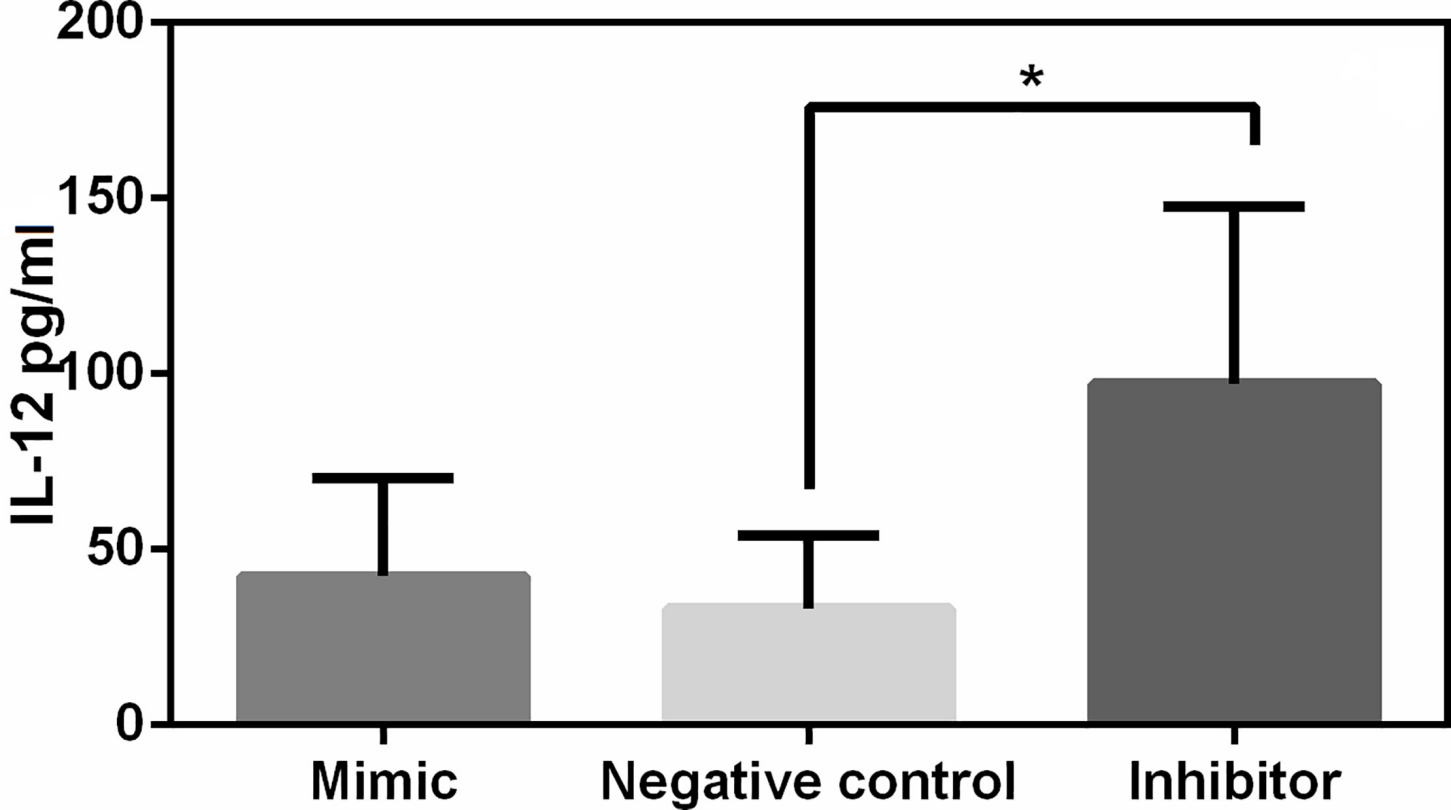
IL-12 production. IL-12 production was quantified in supernatants from SL cultures of dogs transfected with negative control (scrambled), miR 21 mimic, miR 21 inhibitor, all in the presence of Hiperfect (miScript miRNA Mimic and Inhibitor Qiagen, USA), following 67h in culture. SL from dogs naturally infected by *L*. *infantum*. Data represent the mean values of IL-12 + SE. Asterisks represent significance (p<0.05) by Friedman Test with multiple comparison.

Given that IL-12 is an important cytokine for activation of NK and IFN-γ production by T cell, polarization of immune response to Th1 [27] and is induced during CanL [28], we investigated its potential regulation by miR 21, through the use of transfection of miR 21 mimic and inhibitor into SL. We observed an increase in IL-12 in the supernatant of SL cultures of dogs with CanL, following inhibition of miR 21 (Fig 2). In control dogs, SL levels of IL-12 decreased in the supernatant, though not significantly, likely due to low levels of miR 21 in non-infected animals (data not showed).

### Inhibition of miR 21 increases Th1 response

**Fig 3:**
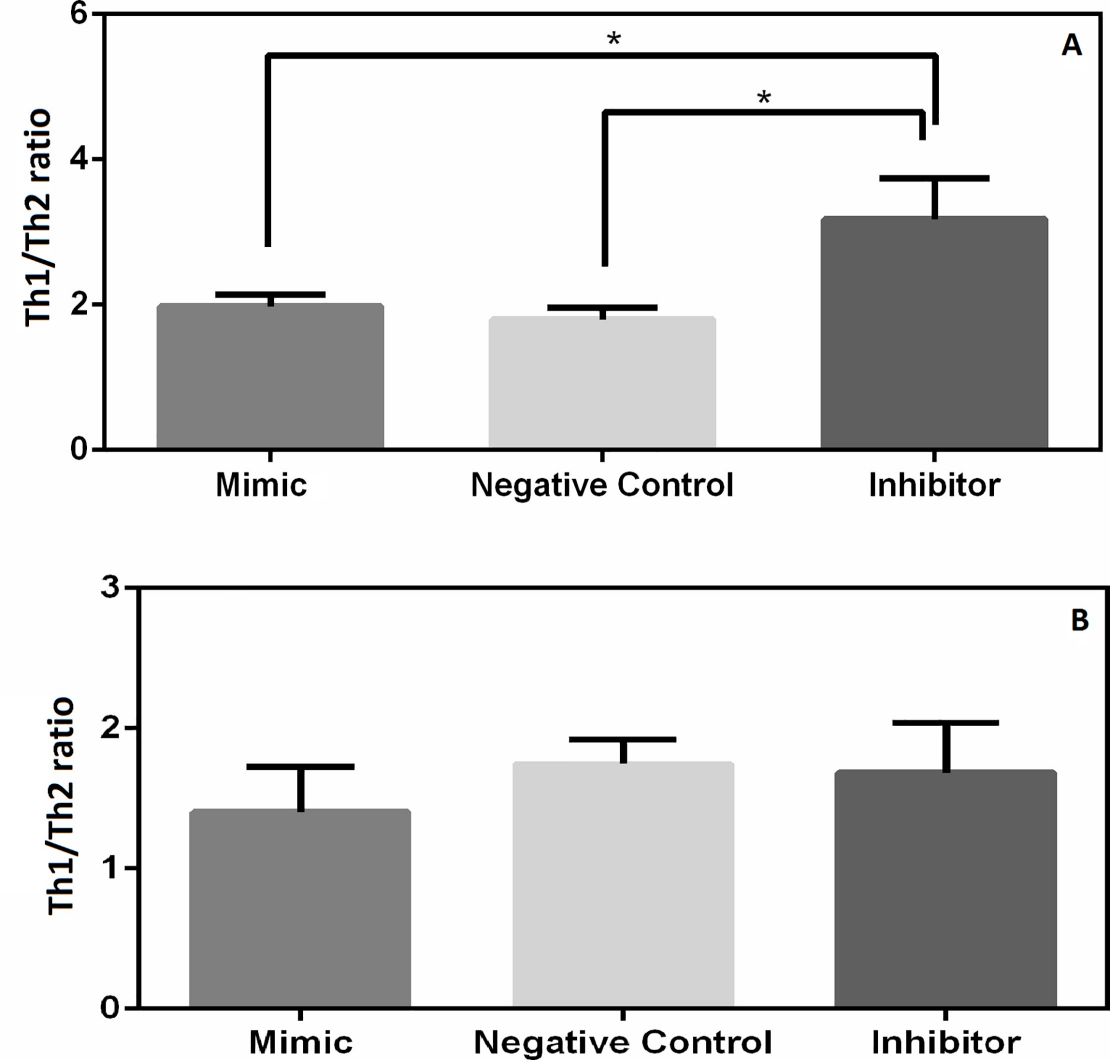
Th1 and Th2 profile. Th1 profile in SL transfected with negative control (scrambled), miR 21 mimic, miR 21 inhibitor and cultured for 67h. (A) SL from dogs with CanL and (B) SL from control non-infected dogs. Data represent the mean values of Th1/Th2 + standard error of the mean. Asterisks represent significance (p<0.05) by Friedman Test with multiple comparison.

To assess whether miR 21 plays a role in the polarization of immune response to Th1 or Th2 in dogs with CanL, T-bet and GATA-3 transcription factors were evaluated (Th1 and Th2 signaling, respectively [29]) following transfection of SL with mimic and inhibitor of miR 21. Inhibition of miR 21 increased Th1 profile population in the SL of dogs with CanL (Fig 3A), whereas in the SL of control dogs, miR 21 mimic resulted in a numerical decrease, albeit not significant, possibly due to low endogenous levels of miR 21 (Fig 3B). Representative images of cytometry analysis were demonstrated in S1 Fig.

### Inhibition of miR 21 decreases parasite load

**Fig 4:**
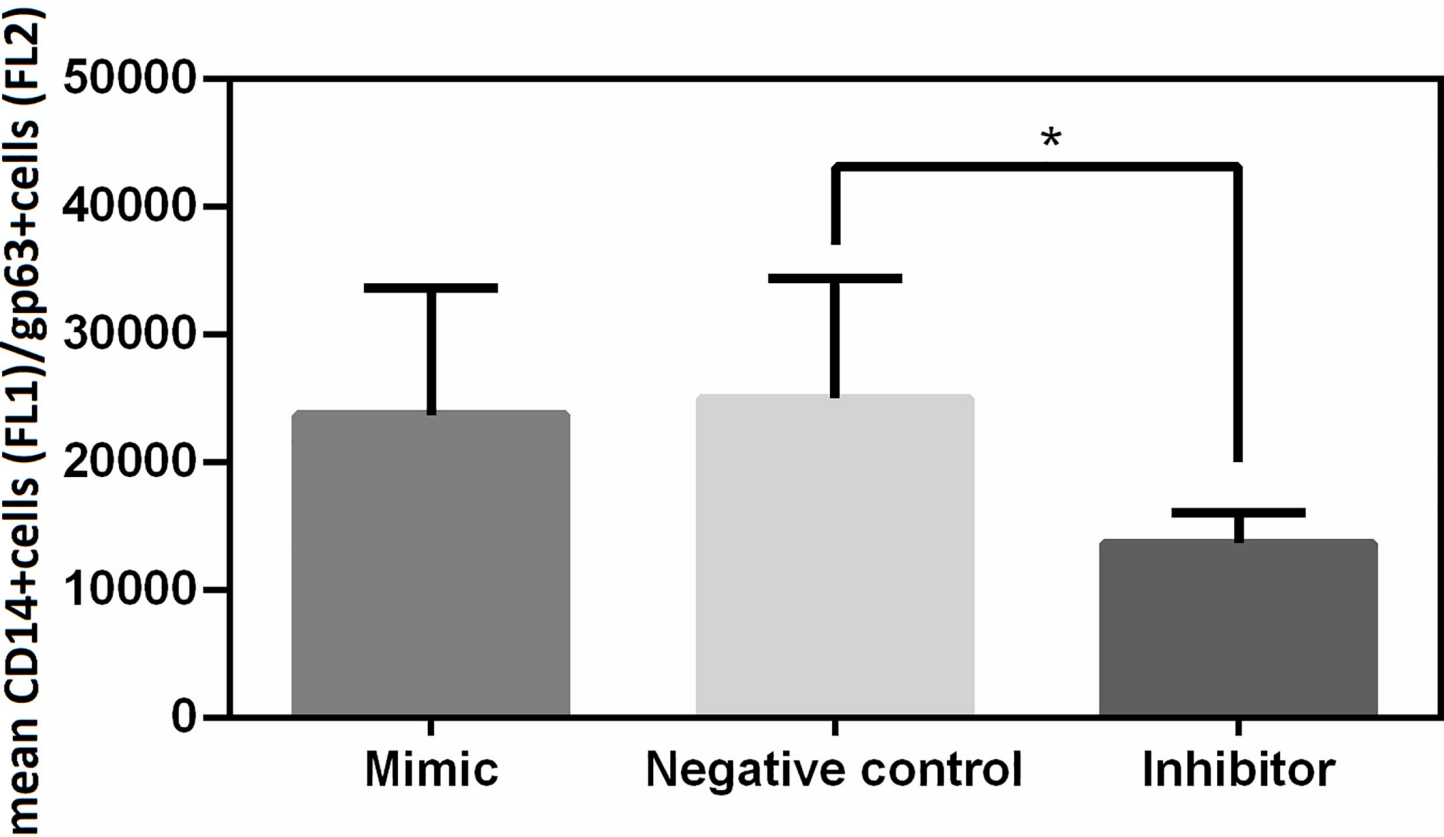
Parasite load. Parasite load in cultures of SL naturally infected by *L*. *infantum* and transfected with negative control (scrambled), miR 21 mimic, miR 21 inhibitor for 67h. Data represent mean + standard error of the mean. Asterisks represent significance (p<0.05) by Friedman Test with multiple comparison.

Parasite load is important in the progression of CanL [30]. To assess whether miR 21 is related to its control, decrease of miR 21 in SL by transfection with miR 21 inhibitor, or increase of miR 21 by transfection of miR 21 mimics was performed. We observed that the inhibition of miR 21 was followed by a decrease of parasite load in SL of dogs with CanL (Fig 4). Representative images of cytometry analysis were demonstrated in S2 Fig.

## Discussion

Following global expression analysis by microarray, we demonstrated that seven miRNAs are differentially expressed in SL of dogs with CanL when compared to control dogs; miR 21, miR 148a, miR 7 and miR 615 showed increased expression, while miR 150, miR 125a and miR 125b showed a decrease in expression. Real-time PCR confirmed increased expression of miR 21, miR 148a and miR 615. Next, the role of miR 21 was evaluated by means of SL cell transfection with mimic and inhibitors, and we could observe modulation of IL-12 cytokine levels, regulation of immune response polarization to the Th1 profile, as well as regulation of parasite load.

MicroRNAs found to be differentially expressed in the SL of dogs with CanL differed from previous studies, conducted in other species. Differential expression of miR 122 was demonstrated in mice macrophages infected by *L*. *infantum* [31], in human macrophages, infected by *L*. *donovani*, members of the miR 30A-3p family were misregulated [15], suggesting that there is variation in miRNA response to *Leishmania* spp. This indicates that it is indispensable to study the dog, given its role as the main reservoir of the disease.

miR 148a showed a significant increase of 2.29 (fold) in SL of dogs with CanL. An exogenous increase in the expression of this miRNA may induce apoptosis in colon cancer cells by silencing Bcl-2 [32]. In dogs with CanL, high levels of apoptosis of CD4 + and CD8 + cells are observed in both blood and spleen, when compared to healthy dogs [33]. Further, miR 148a targets important genes for the regulation of apoptosis, such as FAS and FASLG, suggesting a role for this miRNA in the death of CD4 + and CD8 + cells in dogs with CanL.

Likewise, miR 615 was also increased in SL of dogs with CanL (2.3 fold). This miRNA targets ligand-dependent nuclear corepressor (LCoR), a derepressor of peroxisome proliferator-activated receptor gamma (PPARγ), which promotes the phagocytic capacity of splenic macrophages in mice [34]. In the spleen of dogs with CanL, there is an increase in proliferation and differentiation of macrophages [35]. Therefore, an increase in the expression of this miRNA may increase phagocytic capacity of macrophage in an attempt to combat infection by *L*. *infantum*.

Similarly, miR 21 was significantly increased (3.7 fold) in SL of dogs with CanL. This miRNA is involved with immune regulation as a negative modulator of T cell activation in human [36], and a negative correlation was observed with TNF-α and IL-6 production in human PBMC [37]. In addition, presence of TNF-α is associated with resistance to CanL [12], thus, high levels of miR 21 may be decreasing TNF-α, which could increase splenic parasite load, contributing to disease progression.

In face of the differentially expressed miR 148a, miR 615 and miR 21 in SL from *L*. *infantum* infected dogs, targets were identified in order to search for canonical pathways. Immunity-related pathways were identified such as STAT3, PTEN, cytotoxic T cells mediating apoptosis TGF-β, Th2 activation, Th1 and Th2 activation, p53, crosstalk between dendritic cells and NK, JAK/Stat, p38 MAPK, and antigen presentation, amongst others. Some genes from these pathways have already been described in the disease. In humans with VL, PTEN is actively involved in susceptibility to the disease, its expression is decreased in splenic tissues [38], and similar results were observed in mice macrophages infected with *L*. *major* [39]. Infection of murine macrophages with *L*. *donovani* increases signaling of p38MAPK [40]. *In vitro* infection of human phagocytes with *L*. *donovani* showed that differentially expressed miRNAs interfere with JAK-STAT and TGF-β signaling pathways [14], similarly to the results we presented herein. Therefore, these pathways may be important to guide future studies on immune responses in VL.

Next, we carried out SL cell transfection with mimic and inhibitor of miR 21, due to its involvement in immune response regulation [41]. Inhibition of miR 21 in SL from dogs with CanL showed that miR 21 targets IL-12 cytokine, confirming the involvement of miR 21 in the regulation of IL-12 expression in the canine model. Similar results were observed in mouse dendritic cells following LPS stimulation, where miR 21 deficiency increased IL-12 production [42]. miR 21 binds to the 3´UTR of IL-12p35, which is conserved in several species [43], regulating the production of IL-12. IL-12 is a heterodimeric cytokine produced by most inflammatory cells in response to intracellular pathogens. IL-12 induces the production of IFN-γ by NK cells and T cells, and is important for the maintenance of Th1-type response [44]. In fact, IL-12 neutralization causes amastigote replication in the spleen of BALB/c mice, and IL-12 knockout mice have a greater parasite load in liver and spleen, when compared to wild-type mice [45–47]. In CanL, IL-12 has been observed in the spleen [48–50], but the amount produced seems to be insufficient to maintain a strong Th1-type response, since exogenous IL-12 increases PBMC proliferation and IFN-γ production [48]. High production of miR 21 in dogs with CanL decreases IL-12 production, possibly favoring parasite replication in the spleen.

Moreover, another effect observed by inhibition of miR 21 was the increase in the T-bet / GATA-3 ratio in SL of dogs with CanL. IL-12 signaling induced T-bet transcription in T cells after 24h [51], so the increase in the T-bet/GATA-3 ratio observed could be an indirect action of miR 21 in the polarization of the Th1 response, as previously observed in a model of allergic inflammation in mice [43]. In dogs, the progression of CanL is associated with inhibition of protective Th1 response [11,52], and with an increased non-protective Th2 antibody response. In sick dogs, this may eventually result in tissue damage via different pathomechanisms, notably granulomatous inflammation (e.g. nodular dermatitis, osteomyelitis), immune complex deposition (e.g. glomerulonephritis), and/or autoantibody production (e.g. polymyositis) [53]. Thus, it is feasible that the decrease in miR 21 favored an increase in IL-12, responsible for Th1 polarization in naturally infected dogs.

Furthermore, inhibition of miR 21 also promoted a decrease in parasite load in SL of dogs with CanL. Decreased parasite load has been associated with increased TNF-α, and the asymptomatic condition in dogs seems to depend on differential expression of Th1 cytokines [12]. Increase of miR 21 in mice is associated with the M2 phenotype in macrophages, characterized by high production of IL-10, increased arginase 1 and decreased TNF [41]; on the other hand, deficiency of miR 21 in mice promotes macrophage polarization for M1 [54]. In dogs with CanL, the M2 phenotype predominates in splenic macrophages, where high parasite load is observed [53,55], therefore, it is tempting to suggest that inhibition of miR 21 may have increased the percentage of macrophages with M1 profile, producing nitric oxide and consequently decreasing parasite load.

Besides that, another possibility for the observed decrease in parasite load, following inhibition of miR 21, is related to the activation of T cells. Expression of miR 21 affects ERK phosphorylation and AP-1 activity by inhibiting T cell activation and production of IFN-γ [36]. In mice, dysfunction of cellular immunity with VL has been attributed to dephosphorylation of key molecules involved in signaling, leading to inactivation of T cells [56]. Inhibition of miR 21 may have promoted the reduction of parasite load by restoring the signaling involved in T cell activation, however further studies are needed to confirm this hypothesis in CanL.

Finally, increased production of miR 21, targeting IL-12 and impairing immune response, has also been observed in infection by other intracellular pathogens. Increase of miR 21 induced by vaccination with Bacillus Calmette-Guerin (BCG) in mice suppresses IL-12 production by targeting IL-12p35, impairing anti-mycobacterial T cell responses both *in vitro* and *in vivo* [57]. Brucella Omp25 infection in human macrophages induces miR 21-5p and negatively regulates IL-12 production at both transcriptional and post-transcriptional levels, impairing macrophage function [58]. Taken together, these results suggest that the induction of miR 21 may be involved in an escape mechanism, common to intracellular pathogens in macrophages.

## Conclusion

*L*. *infantum* infection alters expression of miRNAs and miR 21 interferes with cellular immune response in dogs with CanL.

## Supporting information

S1 Table Complete blood count of dogs used for microarray analysis. (DOCX)

S2 Table Biochemical profile of dogs used for microarray analysis. (DOCX)

S3 Table Main clinical signs associated with CanL, serological and molecular diagnosis of dogs with used for microarray analysis. (DOCX)

S4 Table Optical density on ELISA, PCR diagnostic and clinical signs of naturally infected dogs (infected group) and healthy dogs (control group) used for transfection analysis. (DOCX)

S5 Table Complete blood count of infected and control dogs used for transfection analysis. (DOCX)

S6 Table Biochemical profile of infected and control group dogs used for transfection analysis. (DOCX)

S7 Table Canonical pathways predicted for differentially regulated miRNAs in CanL. (DOCX)

S8 Table Top 30—GO Biological Process 2018 (A), GO Cellular Component 2018 (B), GO Molecular Function 2018 (C).
(DOCX)

S1 Fig Representative Histogram obtained from Flow Cytometry analysis of T-Bet (FL1) and GATA-3 transcription factors (FL2) in CanL (n = 8) transfected with Negative control (Scrambled), miR 21 mimic and miR 21 inhibitor, all with the presence of Hiperfect (miScript miRNA Mimic and Inhibitor Qiagen, USA) for 67h. Selected lymphocyte population (A), in the presence of a miR 21 mimic (B), in the presence of a Negative control (scrambled) (C), in the presence of a miR 21 Inhibitor (D). Gate in R is a lymphoid cell mark, gate in M marks T-bet and GATA-3, red peak marks T-bet and GATA-3 positive cells and black peak is positive for their respective isotypes control.
(TIF)

S2 Fig Representative histogram obtained from the CD14+ (FL1) and gp63 (FL2) -labelled flow cytometry analysis on splenic leukocytes from dogs with CanL transfected with miR 21 mimic, negative control (scrambled), and miR 21 inhibitor, all with the presence of Hiperfect (miScript miRNA Mimic and Inhibitor Qiagen, USA) for 67h. (A) Orange peak population labeled with CD14+ (M11), red peak positivity for gp63 and CD14+ cell (B) in the presence of a miR 21 Mimic (C) in the presence of a negative control (scrambled) (D) and in the presence of the Inhibitor of miR 21 (D).
(TIF)

## References

[1] Moreno J, Alvar J. Canine leishmaniasis: Epidemiological risk and the experimental model. Trends Parasitol. 2002;18: 399–405. 10.1016/s1471-4922(02)02347-4 12377257

[2] Murray HW, Berman JD, Davies CR, Saravia NG. Advances in leishmaniasis. Lancet. 2005;366: 1561–1577. 10.1016/S0140-6736(05)67629-5 16257344

[3] Desjeux P. Leishmaniasis: current situation and new perspectives. Comp Immunol Microbiol Infect Dis. 2004;27: 305–318. 10.1016/j.cimid.2004.03.004 15225981

[4] Mancianti F, Gramiccia M, Gradoni L, Pieri S. Studies on canine leishmaniasis control. 1. Evolution of infection of different clinical forms of canine leishmaniasis following antimonial treatment. Trans R Soc Trop Med Hyg. 1988;82: 566–7. Available: http://www.ncbi.nlm.nih.gov/pubmed/3076714 10.1016/0035-9203(88)90510-x 3076714

[5] Badaró R, Jones TC, Lorenço R, Cerf BJ, Sampaio D, Carvalho EM, et al A prospective study of visceral leishmaniasis in an endemic area of Brazil. J Infect Dis. 1986;154: 639–49. 10.1093/infdis/154.4.639 3745974

[6] Semião-Santos SJ, el Harith A, Ferreira E, Pires CA, Sousa C, Gusmão R. Evora district as a new focus for canine leishmaniasis in Portugal. Parasitol Res. 1995;81: 235–9. Available: http://www.ncbi.nlm.nih.gov/pubmed/7770430 10.1007/bf00937115 7770430

[7] Strauss-Ayali D, Baneth G, Jaffe CL. Splenic immune responses during canine visceral leishmaniasis. Vet Res. 2007;38: 547–564. 10.1051/vetres:2007015 17540157

[8] Reis AB, Martins-Filho OA, Teixeira-Carvalho A, Carvalho MG, Mayrink W, França-Silva JC, et al Parasite density and impaired biochemical/hematological status are associated with severe clinical aspects of canine visceral leishmaniasis. Res Vet Sci. 2006;81: 68–75. 10.1016/j.rvsc.2005.09.011 16288789

[9] Reis AB, Martins-Filho OA, Teixeira-Carvalho A, Giunchetti RC, Carneiro CM, Mayrink W, et al Systemic and compartmentalized immune response in canine visceral leishmaniasis. Vet Immunol Immunopathol. 2009;128: 87–95. 10.1016/j.vetimm.2008.10.307 19054576

[10] Maia C, Campino L. Cytokine and Phenotypic Cell Profiles of *Leishmania infantum* Infection in the Dog. J Trop Med. 2012;2012: 1–7. 10.1155/2012/541571 21845197 PMC3154519

[11] Pinelli E, Killick-Kendrick R, Wagenaar J, Bernadina W, del Real G, Ruitenberg J. Cellular and humoral immune responses in dogs experimentally and naturally infected with Leishmania infantum. Infect Immun. American Society for Microbiology; 1994;62: 229–35. Available: http://www.ncbi.nlm.nih.gov/pubmed/8262632 10.1128/iai.62.1.229-235.1994 PMC186091 8262632

[12] Alves CF, de Amorim IFG, Moura EP, Ribeiro RR, Alves CF, Michalick MS, et al Expression of IFN-γ, TNF-α, IL-10 and TGF-β in lymph nodes associates with parasite load and clinical form of disease in dogs naturally infected with Leishmania (Leishmania) chagasi. Vet Immunol Immunopathol. 2009;128: 349–358. 10.1016/j.vetimm.2008.11.020 19124159

[13] Muxel SM, Laranjeira-Silva MF, Zampieri RA, Floeter-Winter LM. Leishmania (Leishmania) amazonensis induces macrophage miR-294 and miR-721 expression and modulates infection by targeting NOS2 and L-arginine metabolism. Sci Rep. 2017;7: 44141 10.1038/srep44141 28276497 PMC5343489

[14] Geraci NS, Tan JC, Mcdowell MA. Characterization of microRNA expression profiles in Leishmania-infected human phagocytes. Parasite Immunol. 2015;37: 43–51. 10.1111/pim.12156 25376316 PMC4287219

[15] Singh AK, Pandey RK, Shaha C, Madhubala R. MicroRNA expression profiling of Leishmania donovani-infected host cells uncovers the regulatory role of MIR30A-3p in host autophagy. Autophagy. 2016; 1–15. 10.1080/15548627.2015.1100356 27459332 PMC5079678

[16] Bragato JP, Melo LM, Venturin GL, Rebech GT, Garcia LE, Lopes FL, et al Relationship of peripheral blood mononuclear cells miRNA expression and parasitic load in canine visceral leishmaniasis. Afrin F, editor. PLoS One. 2018;13: e0206876 10.1371/journal.pone.0206876 30517108 PMC6281177

[17] Tiwari N, Kumar V, Gedda MR, Singh AK, Singh VK, Gannavaram S, et al Identification and Characterization of miRNAs in Response to Leishmania donovani Infection: Delineation of Their Roles in Macrophage Dysfunction. Front Microbiol. Frontiers Media SA; 2017;8: 314 10.3389/fmicb.2017.00314 28303124 PMC5332369

[18] Bragato JP, Melo LM, Venturin GL, Rebech GT, Garcia LE, Lopes FL, et al Data on differentially expressed miRNAs in dogs infected with Leishmania infantum. Data Br. Elsevier; 2018;17: 218–225. 10.1016/J.DIB.2018.01.007 29876389 PMC5988227

[19] Solano-Gallego L, Koutinas A, Miró G, Cardoso L, Pennisi MG, Ferrer L, et al Directions for the diagnosis, clinical staging, treatment and prevention of canine leishmaniosis. Vet Parasitol. 2009;165: 1–18. 10.1016/j.vetpar.2009.05.022 19559536

[20] Lima VMF, Gonçalves ME, Ikeda FA, Luvizotto MCR, Feitosa MM. Anti-leishmania antibodies in cerebrospinal fluid from dogs with visceral leishmaniasis. Braz J Med Biol Res. 2003;36: 485–489. 10.1590/s0100-879x2003000400010 12700826

[21] Sanches L da C, Martini CC de, Nakamura AA, Santiago MEB, Dolabela de Lima B, Lima VMF de, et al Natural canine infection by Leishmania infantum and Leishmania amazonensis and their implications for disease control. Rev Bras Parasitol Veterinária. Colégio Brasileiro de Parasitologia Veterinária; 2016;25: 465–469. 10.1590/s1984-29612016071 27925065

[22] Lima VMF de, Fattori KR, de Souza F, Eugênio FR, Santos PSP dos, Rozza DB, et al Apoptosis in T lymphocytes from spleen tissue and peripheral blood of L. (L.) chagasi naturally infected dogs. Vet Parasitol. 2012;184: 147–153. 10.1016/j.vetpar.2011.08.024 21899954

[23] Ranasinghe S, Rogers ME, Hamilton JGC, Bates PA, Maingon RDC. A real-time PCR assay to estimate Leishmania chagasi load in its natural sand fly vector Lutzomyia longipalpis. Trans R Soc Trop Med Hyg. 2008;102: 875–882. 10.1016/j.trstmh.2008.04.003 18501935 PMC2678673

[24] Chen EY, Tan CM, Kou Y, Duan Q, Wang Z, Meirelles G, et al Enrichr: interactive and collaborative HTML5 gene list enrichment analysis tool. BMC Bioinformatics. 2013;14: 128 10.1186/1471-2105-14-128 23586463 PMC3637064

[25] Kuleshov M V., Jones MR, Rouillard AD, Fernandez NF, Duan Q, Wang Z, et al Enrichr: a comprehensive gene set enrichment analysis web server 2016 update. Nucleic Acids Res. 2016;44: W90–W97. 10.1093/nar/gkw377 27141961 PMC4987924

[26] Di Giorgio C, Ridoux O, Delmas F, Azas N, Gasquet M, Timon-David P. Flow cytometric detection of Leishmania parasites in human monocyte-derived macrophages: application to antileishmanial-drug testing. Antimicrob Agents Chemother. 2000;44: 3074–8. 10.1128/aac.44.11.3074-3078.2000 11036025 PMC101605

[27] Del Vecchio M, Bajetta E, Canova S, Lotze MT, Wesa A, Parmiani G, et al Interleukin-12: Biological Properties and Clinical Application. Clin Cancer Res. 2007;13: 4677–4685. 10.1158/1078-0432.CCR-07-0776 17699845

[28] Diaz S, Fonseca IP da, Rodrigues A, Martins C, Cartaxeiro C, Silva MJ, et al Canine leishmaniosis. Modulation of macrophage/lymphocyte interactions by L. infantum. Vet Parasitol. 2012;189: 137–144. 10.1016/j.vetpar.2012.05.004 22698797

[29] Kanhere A, Hertweck A, Bhatia U, Gökmen MR, Perucha E, Jackson I, et al T-bet and GATA3 orchestrate Th1 and Th2 differentiation through lineage-specific targeting of distal regulatory elements. Nat Commun. Nature Publishing Group; 2012;3: 1268 10.1038/ncomms2260 23232398 PMC3535338

[30] Torrecilha RBP, Utsunomiya YT, Bosco AM, Almeida BF, Pereira PP, Narciso LG, et al Correlations between peripheral parasite load and common clinical and laboratory alterations in dogs with visceral leishmaniasis. Prev Vet Med. 2016;132: 83–87. 10.1016/j.prevetmed.2016.08.006 27664450

[31] Silva SC, Silva DF, Almeida TC, Perasoli FB, da Silva ATP, da Silva GN, et al Behavior of two Leishmania infantum strains-evaluation of susceptibility to antimonials and expression of microRNAs in experimentally infected J774 macrophages and in BALB/c mice. Parasitol Res. 2018;117: 2881–2893. 10.1007/s00436-018-5979-3 29943317

[32] Zhang J, Ying Z, Tang Z, Long L, Li K. MicroRNA-148a Promotes Myogenic Differentiation by Targeting the ROCK1 Gene. J Biol Chem. 2012;287: 21093–21101. 10.1074/jbc.M111.330381 22547064 PMC3375532

[33] Silva KLO, Melo LM, Perosso J, Oliveira BB, Santos PSP dos, Eugênio F de R, et al CD95 (FAS) and CD178 (FASL) induce the apoptosis of CD4+ and CD8+ cells isolated from the peripheral blood and spleen of dogs naturally infected with Leishmania spp. Vet Parasitol. 2013;197: 470–476. 10.1016/j.vetpar.2013.07.012 23920055

[34] Jiang A, Zhang S, Li Z, Liang R, Ren S, Li J, et al miR-615-3p promotes the phagocytic capacity of splenic macrophages by targeting ligand-dependent nuclear receptor corepressor in cirrhosis-related portal hypertension. Exp Biol Med. 2011;236: 672–680. 10.1258/ebm.2011.010349 21565892

[35] Alexandre-Pires G, Pais D, Correia M, Pina JAE. Leishmaniosis—a report about the microvascular and cellular architecture of the infected spleen in Canis familiaris. Microsc Res Tech. 2006;69: 227–35. 10.1002/jemt.20267 16586484

[36] Carissimi C, Carucci N, Colombo T, Piconese S, Azzalin G, Cipolletta E, et al miR-21 is a negative modulator of T-cell activation. Biochimie. 2014;107: 319–326. 10.1016/j.biochi.2014.09.021 25304039

[37] Mazloom H, Alizadeh S, Esfahani EN, Razi F, Meshkani R. Decreased expression of microRNA-21 is associated with increased cytokine production in peripheral blood mononuclear cells (PBMCs) of obese type 2 diabetic and non-diabetic subjects. Mol Cell Biochem. 2016;419: 11–17. 10.1007/s11010-016-2743-9 27370645

[38] Sudarshan M, Singh T, Singh B, Chakravarty J, Sundar S. Suppression of host PTEN gene expression for Leishmania donovani survival in Indian visceral leishmaniasis. Microbes Infect. 2016;18: 369–72. 10.1016/j.micinf.2015.12.008 26774334 PMC4860145

[39] Kuroda S, Nishio M, Sasaki T, Horie Y, Kawahara K, Sasaki M, et al Effective clearance of intracellular Leishmania major in vivo requires Pten in macrophages. Eur J Immunol. 2008;38: 1331–40. 10.1002/eji.200737302 18398930

[40] Liu L, Wang L, Zhao Y, Wang Y, Wang Z, Qiao Z. Testosterone attenuates p38 MAPK pathway during Leishmania donovani infection of macrophages. Parasitol Res. 2006;99: 189–193. 10.1007/s00436-006-0168-1 16547729

[41] Sheedy FJ. Turning 21: Induction of miR-21 as a Key Switch in the Inflammatory Response. Front Immunol. 2015;6 10.3389/fimmu.2015.00019 25688245 PMC4310327

[42] Lu TX, Hartner J, Lim E-J, Fabry V, Mingler MK, Cole ET, et al MicroRNA-21 Limits In Vivo Immune Response-Mediated Activation of the IL-12/IFN- Pathway, Th1 Polarization, and the Severity of Delayed-Type Hypersensitivity. J Immunol. 2011;187: 3362–3373. 10.4049/jimmunol.1101235 21849676 PMC3175642

[43] Lu TX, Munitz A, Rothenberg ME. MicroRNA-21 is up-regulated in allergic airway inflammation and regulates IL-12p35 expression. J Immunol. American Association of Immunologists; 2009;182: 4994–5002. 10.4049/jimmunol.0803560 19342679 PMC4280862

[44] Seder RA, Gazzinelli R, Sher A, Paul WE. Interleukin 12 acts directly on CD4+ T cells to enhance priming for interferon gamma production and diminishes interleukin 4 inhibition of such priming. Proc Natl Acad Sci U S A. 1993;90: 10188–92. 10.1073/pnas.90.21.10188 7901851 PMC47739

[45] Engwerda CR, Murphy ML, Cotterell SEJ, Smelt SC, Kaye PM. Neutralization of IL-12 demonstrates the existence of discrete organ-specific phases in the control ofLeishmania donovani. Eur J Immunol. 1998;28: 669–680. 10.1002/(SICI)1521-4141(199802)28:02<669::AID-IMMU669>3.0.CO;2-N 9521077

[46] Murray HW, Montelibano C, Peterson R, Sypek JP. Interleukin‐12 Regulates the Response to Chemotherapy in Experimental Visceral Leishmaniasis. J Infect Dis. 2000;182: 1497–1502. 10.1086/315890 11023473

[47] Satoskar AR, Rodig S, Telford SR, Satoskar AA, Ghosh SK, von Lichtenberg F, et al IL-12 gene-deficient C57BL/6 mice are susceptible to Leishmania donovani but have diminished hepatic immunopathology. Eur J Immunol. 2000;30: 834–9. 10.1002/1521-4141(200003)30:3<834::AID-IMMU834>3.0.CO;2-9 10741399

[48] Strauss-Ayali D, Baneth G, Shor S, Okano F, Jaffe CL. Interleukin-12 augments a Th1-type immune response manifested as lymphocyte proliferation and interferon gamma production in Leishmania infantum-infected dogs. Int J Parasitol. 2005;35: 63–73. 10.1016/j.ijpara.2004.10.015 15619517

[49] Santos-Gomes GM, Rosa R, Leandro C, Cortes S, Romão P, Silveira H. Cytokine expression during the outcome of canine experimental infection by Leishmania infantum. Vet Immunol Immunopathol. 2002;88: 21–30. Available: http://www.ncbi.nlm.nih.gov/pubmed/12088641 10.1016/s0165-2427(02)00134-4 12088641

[50] Lage RS, Oliveira GC, Busek SU, Guerra LL, Giunchetti RC, Corrêa-Oliveira R, et al Analysis of the cytokine profile in spleen cells from dogs naturally infected by Leishmania chagasi. Vet Immunol Immunopathol. 2007;115: 135–145. 10.1016/j.vetimm.2006.10.001 17097741

[51] Ylikoski E, Lund R, Kyläniemi M, Filén S, Kilpeläinen M, Savolainen J, et al IL-12 up-regulates T-bet independently of IFN-γ in human CD4+ T cells. Eur J Immunol. 2005;35: 3297–3306. 10.1002/eji.200526101 16220539

[52] Pinelli E, Gonzalo RM, Boog CJ, Rutten VP, Gebhard D, del Real G, et al Leishmania infantum-specific T cell lines derived from asymptomatic dogs that lyse infected macrophages in a major histocompatibility complex-restricted manner. Eur J Immunol. 1995;25: 1594–600. 10.1002/eji.1830250619 7614987

[53] Koutinas AF, Koutinas CK. Pathologic Mechanisms Underlying the Clinical Findings in Canine Leishmaniosis due to *Leishmania infantum/chagasi*. Vet Pathol. 2014;51: 527–538. 10.1177/0300985814521248 24510947

[54] Xi J, Huang Q, Wang L, Ma X, Deng Q, Kumar M, et al miR-21 depletion in macrophages promotes tumoricidal polarization and enhances PD-1 immunotherapy. Oncogene. NIH Public Access; 2018;37: 3151–3165. 10.1038/s41388-018-0178-3 29540832 PMC5993583

[55] Moreira PRR, Fernando FS, Montassier HJ, André MR, de Oliveira Vasconcelos R. Polarized M2 macrophages in dogs with visceral leishmaniasis. Vet Parasitol. 2016;226 10.1016/j.vetpar.2016.06.032 27514887

[56] Mukherjee P, Sen PC, Ghose AC. Lymph node cells from BALB/c mice with chronic visceral leishmaniasis exhibiting cellular anergy and apoptosis: involvement of Ser/Thr phosphatase. Apoptosis. 2006;11: 2013–29. 10.1007/s10495-006-0088-7 17013755

[57] Wu Z, Lu H, Sheng J, Li L. Inductive microRNA-21 impairs anti-mycobacterial responses by targeting IL-12 and Bcl-2. FEBS Lett. 2012;586: 2459–2467. 10.1016/j.febslet.2012.06.004 22710123

[58] Cui B, Liu W, Wang X, Chen Y, Du Q, Zhao X, et al Brucella Omp25 Upregulates miR-155, miR-21-5p, and miR-23b to Inhibit Interleukin-12 Production via Modulation of Programmed Death-1 Signaling in Human Monocyte/Macrophages. Front Immunol. 2017;8: 708 10.3389/fimmu.2017.00708 28694807 PMC5483987

