# Supplementary material for: Induction of miR 21 impairs the anti-Leishmania response through inhibition of IL-12 in canine splenic leukocytes
Source: PLoS One. 2019 Dec 11;14(12):e0226192. doi: 10.1371/journal.pone.0226192 (PMC6905561; doi:10.1371/journal.pone.0226192)
Supplement: S1 Table — (DOCX) [file pone.0226192.s001.docx]

**S1 Table.** Complete blood count of dogs used for microarray analysis.

| Animal | RBC | VG | Hemoglobin | VCM | CHCM | Wbc | Segmented | Lymphocyte | Monocyte | Eosinophil | Basophil | Plt | Tpp |
| --- | --- | --- | --- | --- | --- | --- | --- | --- | --- | --- | --- | --- | --- |
| Reference value | 5.5 - 8.5 x10^6^/µL | 37 – 55 % | 12—18 g/dL | 60 – 77 fL | 32 – 36 % | 6–17 x10³/µL | 3.000- 11.000/µL | 1.000- 4.800/µL | 150- 1.350/µL | 150- 1.250/µL | Rares/µL | 160– 430x10³/µL | 6.0-8.0 g/dL |
| Control 1 | 8.26 | 55 | 18 | 66.58 | 32.72 | 10.2 | 6.324 | 3.468 | 102 | 306 | 0 | 341 | 5.2 |
| Control 2 | 7.21 | 53.8 | 18.8 | 74.7 | 34.9 | 13.1 | 6.419 | 6.668 | 174 | 261 | 0 | 341 | 5.2 |
| Control 3 | 5.32 | 41.2 | 12.9 | 77.5 | 31.3 | 6.4 | 4.480 | 1.024 | 512 | 384 | 0 | 211 | 6 |
| Control 4 | 7.43 | 56.7 | 20.4 | 76.4 | 35.9 | 10.6 | 7.844 | 1.060 | 424 | 1.250 | 0 | 228 | 7 |
| Infected 1 | 5.05 | 33 | 11.5 | 65.35 | 34.85 | 2.5 | 9.500 | 1.375 | 1.125 | 500 | 0 | 420 | 9 |
| Infected 2 | 4.91 | 31 | 11.1 | 63.14 | 35.81 | 11.8 | 10.738 | 708 | 354 | 0 | 0 | 60 | 10.9 |
| Infected 3 | 3.41 | 25.4 | 8.6 | 74.5 | 25.2 | 10.2 | 7.752 | 1.836 | 510 | 102 | 0 | 82 | 9 |
| Infected 4 | 5.27 | 34.5 | 10.4 | 65.6 | 30.1 | 6.3 | 4.158 | 1.071 | 756 | 63 | 0 | 159 | 6.8 |
| Infected 5 | 4.24 | 31.3 | 10.6 | 74 | 33.8 | 14.8 | 6.808 | 5.032 | 2.220 | 740 | 0 | 168 | 11 |
| Infected 6 | 4.93 | 31.2 | 10.3 | 63.4 | 33 | 14.9 | 9.238 | 3.427 | 1.043 | 1.192 | 0 | 277 | 10.2 |
| Infected 7 | 4.23 | 33 | 10.3 | 63.5 | 33.1 | 12.5 | 10.000 | 1.750 | 375 | 375 | 0 | 331 | 8 |
| Infected 8 | 5.28 | 34 | 10.6 | 60.2 | 32.4 | 9.8 | 6.272 | 2.156 | 392 | 980 | 0 | 145 | 9 |

RBC (red blood cells) CHCM (mean corpuscular hemoglobin concentration) MCV (mean corpuscular volume), TPP (total plasma protein)
