# Supplementary material for: Induction of miR 21 impairs the anti-Leishmania response through inhibition of IL-12 in canine splenic leukocytes
Source: PLoS One. 2019 Dec 11;14(12):e0226192. doi: 10.1371/journal.pone.0226192 (PMC6905561; doi:10.1371/journal.pone.0226192)
Supplement: S3 Table — (DOCX) [file pone.0226192.s003.docx]

**S3 Table.** Main clinical signs associated with CanL, serological and molecular diagnosis of dogs with used for microarray analysis.

| **Animal** | **ELISA (O.D.)** | **PCR (parasites/5x10^6^ cells)** | **Clinical Signs** |
| --- | --- | --- | --- |
| Control 1 | 0.026 | 0,0 | No clinical signs |
| Control 2 | 0.028 | 0,0 | No clinical signs |
| Control 3 | 0.049 | 0,0 | No clinical signs |
| Control 4 | 0.055 | 0,0 | No clinical signs |
| Infected 1 | 1.333 | 14607 | Onychogrifose, skin lesion, alopecia, ear lesion, anemia, hepatosplenomegaly |
| Infected 2 | 0.511 | 27202 | Onychogrifose, cachexia, skin lesion, alopecia, ear lesion, anemia, hepatosplenomegaly |
| Infected 3 | 0.974 | 133000 | Onychogrifose, cachexia, ear lesion, anemia |
| Infected 4 | 1.283 | 284000 | Onychogrifose, alopecia, ear lesion, anemia |
| Infected 5 | 0.356 | 274000 | Lymphadenopathy, Onychogrifose, skin lesion, hepatosplenomegaly |
| Infected 6 | 1.323 | 222000 | Onychogrifose, skin lesion, ear lesion, anemia |
| Infected 7 | 1.315 | 108000 | Onychogrifosis, cachexia, skin lesion, ear lesion, anemia |
| Infected 8 | 0.907 | 1900000 | Onychogrifose, cachexia, ear lesion, anemia, hepatosplenomegaly |

O.D.: Optical density.
