# Supplementary material for: Induction of miR 21 impairs the anti-Leishmania response through inhibition of IL-12 in canine splenic leukocytes
Source: PLoS One. 2019 Dec 11;14(12):e0226192. doi: 10.1371/journal.pone.0226192 (PMC6905561; doi:10.1371/journal.pone.0226192)
Supplement: S4 Table — (DOCX) [file pone.0226192.s004.docx]

**S4 Table**. Optical density on ELISA, PCR diagnostic and clinical signs of naturally infected dogs (infected group) and healthy dogs (control group) used for transfection analysis.

| **Animal** | **ELISA** | **PCR** | **Clinical Signs** |
| --- | --- | --- | --- |
| Control 1 | 0.046 | Negative | No clinical signs |
| Control 2 | 0.047 | Negative | No clinical signs |
| Control 3 | 0.048 | Negative | No clinical signs |
| Control 4 | 0.056 | Negative | No clinical signs |
| Infected 1 | 1.044 | Positive | Lymphadenopathy, Ear lesions, Periocular lesions |
| Infected 2 | 1.124 | Positive | Lymphadenopathy, Onychogrifose, Ear lesions, Periocular lesions |
| Infected 3 | 1.030 | Positive | Lymphoadenopathy, Onychogrifose, Ear lesions |
| Infected 4 | 0.479 | Positive | Ear lesions, Periocular lesions, Ascites, Fucking lesion. |
| Infected 5 | 0.441 | Positive | Lymphadenopathy, Ear lesions, Periocular lesions |
| Infected 6 | 1.103 | Positive | Fracture Lesion, Lymphadenopathy, Ear lesions |
| Infected 7 | 0.506 | Positive | Lymphadenopathy, Hepatosplenomegaly, Ear lesions |
| Infected 8 | 0.924 | Positive | Lymphadenopathy, Onychogrifose, Cachexia |
