# Supplementary material for: Induction of miR 21 impairs the anti-Leishmania response through inhibition of IL-12 in canine splenic leukocytes
Source: PLoS One. 2019 Dec 11;14(12):e0226192. doi: 10.1371/journal.pone.0226192 (PMC6905561; doi:10.1371/journal.pone.0226192)
Supplement: S5 Table — (DOCX) [file pone.0226192.s005.docx]

| Animal | RBC | GV | Hemoglobin | VCM | CHCM | Leukocyte | Neutrophil | Lymphocyte | Monocyte | Eosinophil | Plt |
| --- | --- | --- | --- | --- | --- | --- | --- | --- | --- | --- | --- |
| Reference value | 5.5 - 8.5 x10^6^/µL | 37 – 55 % | 12—18 g/dL | 60 – 77 fL | 32 – 36 % | 6 – 17 x10³/ µL | 3000 - 1000/µL | 1000 - 4800/µL | 150 - 1350/µL | 150 - 1250/µL | 160 – 430x10³/µL |
| Control 1 | 8.6 | 58.6 | 21.6 | 67.5 | 36.9 | 14.26 | 6.702 | 4.535 | 528 | 2.481 | 201 |
| Control 2 | 8.2 | 56.3 | 20.6 | 66.1 | 36.5 | 14.49 | 8.259 | 4.970 | 725 | 435 | 256 |
| Control 3 | 7.4 | 53.5 | 19.6 | 67.3 | 36.6 | 10.64 | 6.065 | 2.554 | 532 | 1.373 | 439 |
| Control 4 | 5.77 | 40.5 | 14.7 | 70.2 | 36.4 | 20.22 | 11.222 | 5.540 | 849 | 2.608 | 386 |
| Infected 1 | 4.64 | 29.0 | 9.4 | 62.5 | 32.4 | 6.3 | 7500 | 1800 | 400 | 300 | 67 |
| Infected 2 | 4.45 | 32.3 | 11.2 | 72.7 | 34.6 | 4.8 | 7000 | 2500 | 400 | 100 | 53 |
| Infected 3 | 5.82 | 32.5 | 10.7 | 55.9 | 32.9 | 32.2 | 7400 | 1600 | 700 | 300 | 227 |
| Infected 4 | 2.39 | 15.5 | 5.2 | 65.0 | 33.5 | 7.5 | 5300 | 3500 | 800 | 400 | 36 |
| Infected 5 | 2.99 | 18.4 | 5.6 | 61.6 | 30.4 | 1.8 | 6400 | 2400 | 1200 | 0 | 40 |
| Infected 6 | 6.52 | 52.2 | 18.1 | 80.2 | 34.6 | 24.1 | 7000 | 2200 | 600 | 200 | 274 |
| Infected 7 | 6.31 | 45.7 | 16.3 | 72.5 | 35.6 | 15.9 | 7500 | 2700 | 800 | 500 | 296 |
| Infected 8 | 5.94 | 35.6 | 11.7 | 60.0 | 32.8 | 6.1 | 7300 | 2200 | 200 | 300 | 136 |

**S5 Table** Complete blood count of infected and control dogs used for transfection analysis.

Abbreviations: RBC (red blood cells) CHCM (mean corpuscular hemoglobin concentration) MCV (mean corpuscular volume)
