# Supplementary material for: Induction of miR 21 impairs the anti-Leishmania response through inhibition of IL-12 in canine splenic leukocytes
Source: PLoS One. 2019 Dec 11;14(12):e0226192. doi: 10.1371/journal.pone.0226192 (PMC6905561; doi:10.1371/journal.pone.0226192)
Supplement: S6 Table — (DOCX) [file pone.0226192.s006.docx]

**S6 Table**. Biochemical profile of infected and control group dogs used for transfection analysis.

| Animal | ALT | AST | Creatinine | ALP | Urea |
| --- | --- | --- | --- | --- | --- |
| Reference value | 21 – 102 UI/L | 23 – 66 UI/L | 0.5 - 1.5 mg/dL | 20 – 156 UI/L | 1.67-8.33 mmol/L |
| Control 1 | 28.41 | 22.04 | 0.78 | 92.93 | 6.32 |
| Control 2 | 35.82 | 34.56 | 1.12 | 143.09 | 7.1 |
| Control 3 | 82.29 | 29.87 | 0.91 | 158.86 | 4.94 |
| Control 4 | 89.19 | 40.77 | 0.91 | 70.53 | 4.95 |
| Infected 1 | 15,05 | 63,88 | 3,30 | 116,09 | 39,79 |
| Infected 2 | 52,92 | 56,83 | 0,57 | 113,3 | 4,31 |
| Infected 3 | 102,85 | 67,11 | 0,59 | 101,71 | 7,18 |
| Infected 4 | 99,03 | 68,01 | 0,83 | 185,19 | 6,55 |
| Infected 5 | 31,39 | 75,53 | 1,03 | 148,84 | 6,15 |
| Infected 6 | 34,70 | 35,56 | 0,73 | 91 | 6,48 |
| Infected 7 | 26,39 | 88,22 | 0,59 | 79,66 | 4,56 |
| Infected 8 | 24,06 | 62,64 | 0,98 | 0 | 6,90 |

ALT (Alanina aminotransferase) AST (aspartato aminotransferase)
