# Supplementary material for: Induction of miR 21 impairs the anti-Leishmania response through inhibition of IL-12 in canine splenic leukocytes
Source: PLoS One. 2019 Dec 11;14(12):e0226192. doi: 10.1371/journal.pone.0226192 (PMC6905561; doi:10.1371/journal.pone.0226192)
Supplement: S7 Table — (DOCX) [file pone.0226192.s007.docx]

**S7 Table.** Canonical pathways predicted for differentially regulated miRNAs in CanL.

| **Ingenuity Canonical Pathways** | **P-value** |
| --- | --- |
| Neuregulin Signaling | 0.0001 |
| STAT3 Pathway | 0.0010 |
| PTEN Signaling | 0.0010 |
| HER-2 Signaling in Breast Cancer | 0.0015 |
| Myc Mediated Apoptosis Signaling | 0.0015 |
| Glioma Signaling | 0.0018 |
| Cytotoxic T Lymphocyte-mediated Apoptosis of Target Cells | 0.0019 |
| Neurotrophin/TRK Signaling | 0.0019 |
| ErbB Signaling | 0.0019 |
| Cholecystokinin/Gastrin-mediated Signaling | 0.0021 |
| Type I Diabetes Mellitus Signaling | 0.0026 |
| TGF-β Signaling | 0.0032 |
| VEGF Family Ligand-Receptor Interactions | 0.0032 |
| Molecular Mechanisms of Cancer | 0.0038 |
| Th2 Pathway | 0.0056 |
| VEGF Signaling | 0.0070 |
| Th1 and Th2 Activation Pathway | 0.0070 |
| Graft-versus-Host Disease Signaling | 0.0070 |
| p53 Signaling | 0.0070 |
| Prolactin Signaling | 0.0070 |
| Crosstalk between Dendritic Cells and Natural Killer Cells | 0.0070 |
| JAK/Stat Signaling | 0.0070 |
| Cell Cycle: G2/M DNA Damage Checkpoint Regulation | 0.0077 |
| Virus Entry via Endocytic Pathways | 0.0085 |
| HGF Signaling | 0.0100 |
| p38 MAPK Signaling | 0.0102 |
| ErbB2-ErbB3 Signaling | 0.0104 |
| Antigen Presentation Pathway | 0.0112 |
| Fc Epsilon RI Signaling | 0.0112 |
| NGF Signaling | 0.0112 |
| ErbB4 Signaling | 0.0112 |
| Axonal Guidance Signaling | 0.0112 |
| Prostate Cancer Signaling | 0.0123 |
| Renin-Angiotensin Signaling | 0.0123 |
| BMP signaling pathway | 0.0138 |
| Melanoma Signaling | 0.0141 |
| Non-Small Cell Lung Cancer Signaling | 0.0141 |
| Hepatic Fibrosis / Hepatic Stellate Cell Activation | 0.0141 |
| IL-6 Signaling | 0.0147 |
| Allograft Rejection Signaling | 0.0147 |
| SAPK/JNK Signaling | 0.0154 |
| Erythropoietin Signaling | 0.0162 |
| Chronic Myeloid Leukemia Signaling | 0.0165 |
| Renal Cell Carcinoma Signaling | 0.0165 |
| Role of NFAT in Cardiac Hypertrophy | 0.0169 |
| Aryl Hydrocarbon Receptor Signaling | 0.0177 |
| Cell Cycle: G1/S Checkpoint Regulation | 0.0177 |
| Human Embryonic Stem Cell Pluripotency | 0.0186 |
| FLT3 Signaling in Hematopoietic Progenitor Cells | 0.0186 |
| PEDF Signaling | 0.0186 |
| Death Receptor Signaling | 0.0186 |
| Autoimmune Thyroid Disease Signaling | 0.0186 |
| Xenobiotic Metabolism Signaling | 0.0186 |
| Paxillin Signaling | 0.0186 |
| Endometrial Cancer Signaling | 0.0186 |
| ERK/MAPK Signaling | 0.0186 |
| Bladder Cancer Signaling | 0.0186 |
| Insulin Receptor Signaling | 0.0186 |
| UVC-Induced MAPK Signaling | 0.0186 |
| Thrombopoietin Signaling | 0.0186 |
| IL-4 Signaling | 0.0186 |
| Apoptosis Signaling | 0.0194 |
| Hereditary Breast Cancer Signaling | 0.0194 |
| T Helper Cell Differentiation | 0.0204 |
| Breast Cancer Regulation by Stathmin1 | 0.0208 |
| Neuroinflammation Signaling Pathway | 0.0239 |
| NF-κB Signaling | 0.0257 |
| VDR/RXR Activation | 0.0263 |
| PI3K/AKT Signaling | 0.0269 |
| Melanocyte Development and Pigmentation Signaling | 0.0269 |
| FAK Signaling | 0.0275 |
| Colorectal Cancer Metastasis Signaling | 0.0281 |
| Regulation of eIF4 and p70S6K Signaling | 0.0295 |
| PAK Signaling | 0.0295 |
| IL-15 Signaling | 0.0302 |
| CCR3 Signaling in Eosinophils | 0.0302 |
| p70S6K Signaling | 0.0331 |
| Glioblastoma Multiforme Signaling | 0.0331 |
| Protein Kinase A Signaling | 0.0338 |
| Small Cell Lung Cancer Signaling | 0.0338 |
| Mouse Embryonic Stem Cell Pluripotency | 0.0338 |
| IGF-1 Signaling | 0.0338 |
| Actin Cytoskeleton Signaling | 0.0338 |
| Inhibition of Matrix Metalloproteases | 0.0338 |
| Actin Nucleation by ARP-WASP Complex | 0.0354 |
| Macropinocytosis Signaling | 0.0354 |
| Oncostatin M Signaling | 0.0354 |
| IL-3 Signaling | 0.0380 |
| Acute Phase Response Signaling | 0.0389 |
| IL-12 Signaling and Production in Macrophages | 0.0389 |
| Altered T Cell and B Cell Signaling in Rheumatoid Arthritis | 0.0389 |
| Cardiac Hypertrophy Signaling | 0.0389 |
| FGF Signaling | 0.0398 |
| IL-7 Signaling Pathway | 0.0398 |
| UVB-Induced MAPK Signaling | 0.0416 |
| G Beta Gamma Signaling | 0.0416 |
| Osteoarthritis Pathway | 0.0416 |
| LPS-stimulated MAPK Signaling | 0.0426 |
| Tumoricidal Function of Hepatic Natural Killer Cells | 0.0457 |
| CCR5 Signaling in Macrophages | 0.0457 |
| HIF1α Signaling | 0.0467 |
| PDGF Signaling | 0.0467 |
| CNTF Signaling | 0.0478 |
| IL-2 Signaling | 0.0478 |
| Thyroid Cancer Signaling | 0.0478 |
| Synaptic Long Term Potentiation | 0.0478 |
| Caveolar-mediated Endocytosis Signaling | 0.0489 |
| RAR Activation | 0.0489 |
| Ceramide Signaling | 0.0489 |
| Natural Killer Cell Signaling | 0.0489 |
| ERK5 Signaling | 0.0489 |
| MSP-RON Signaling Pathway | 0.0489 |
| Antiproliferative Role of TOB in T Cell Signaling | 0.0489 |
| Estrogen-mediated S-phase Entry | 0.0489 |
