# Supplementary material for: Induction of miR 21 impairs the anti-Leishmania response through inhibition of IL-12 in canine splenic leukocytes
Source: PLoS One. 2019 Dec 11;14(12):e0226192. doi: 10.1371/journal.pone.0226192 (PMC6905561; doi:10.1371/journal.pone.0226192)
Supplement: S8 Table — Top 30—GO Biological Process 2018 (A), GO Cellular Component 2018 (B), GO Molecular Function 2018 (C). (DOCX) [file pone.0226192.s008.docx]

**S8a Table.** Top 30 - GO Biological Process 2018

| **Index** | **Name** | **P-value** | **Adjusted p-value** | **Z-score** | **Combined score** |
| --- | --- | --- | --- | --- | --- |
| 1 | positive regulation of protein phosphorylation (GO:0001934) | 2.391e-9 | 0.000001316 | -1.91 | 37.85 |
| 2 | regulation of apoptotic process (GO:0042981) | 9.482e-9 | 0.000003480 | -1.79 | 33.04 |
| 3 | cytokine-mediated signaling pathway (GO:0019221) | 2.667e-11 | 2.937e-8 | -1.35 | 32.76 |
| 4 | transmembrane receptor protein tyrosine kinase signaling pathway (GO:0007169) | 1.955e-8 | 0.000005067 | -1.74 | 30.85 |
| 5 | epidermal growth factor receptor signaling pathway (GO:0007173) | 7.638e-7 | 0.00005947 | -2.15 | 30.29 |
| 6 | antigen processing and presentation of exogenous peptide antigen via MHC class I, TAP-independent (GO:0002480) | 0.000002884 | 0.0001671 | -2.31 | 29.46 |
| 7 | regulation of cyclin-dependent protein kinase activity (GO:1904029) | 6.604e-8 | 0.000009088 | -1.72 | 28.43 |
| 8 | artery development (GO:0060840) | 0.00001610 | 0.0005909 | -2.39 | 26.39 |
| 9 | positive regulation of apoptotic process (GO:0043065) | 2.772e-7 | 0.00002775 | -1.68 | 25.33 |
| 10 | negative regulation of cell junction assembly (GO:1901889) | 0.00002682 | 0.0008204 | -2.34 | 24.65 |
| 11 | regulation of endothelial cell development (GO:1901550) | 0.0001780 | 0.002702 | -2.85 | 24.59 |
| 12 | vascular endothelial growth factor receptor signaling pathway (GO:0048010) | 6.267e-8 | 0.000009088 | -1.44 | 23.94 |
| 13 | ERBB signaling pathway (GO:0038127) | 1.388e-7 | 0.00001528 | -1.49 | 23.49 |
| 14 | positive regulation of cell proliferation (GO:0008284) | 4.189e-7 | 0.00003844 | -1.55 | 22.76 |
| 15 | outflow tract septum morphogenesis (GO:0003148) | 0.00004141 | 0.001036 | -2.25 | 22.73 |
| 16 | extrinsic apoptotic signaling pathway (GO:0097191) | 4.822e-8 | 0.000008848 | -1.34 | 22.53 |
| 17 | negative regulation of ERBB signaling pathway (GO:1901185) | 0.000008489 | 0.0003462 | -1.85 | 21.63 |
| 18 | negative regulation of T-helper cell differentiation (GO:0045623) | 0.0003039 | 0.003792 | -2.67 | 21.58 |
| 19 | axonogenesis (GO:0007409) | 0.000004187 | 0.0002195 | -1.69 | 20.87 |
| 20 | negative regulation of cyclin-dependent protein serine/threonine kinase activity involved in G1/S transition of mitotic cell cycle (GO:0031658) | 0.0002368 | 0.003303 | -2.49 | 20.78 |
| 21 | peripheral nervous system development (GO:0007422) | 0.00004141 | 0.001036 | -2.05 | 20.65 |
| 22 | negative regulation of transcription by competitive promoter binding (GO:0010944) | 0.0003792 | 0.004260 | -2.60 | 20.45 |
| 23 | activation of MAPKKK activity (GO:0000185) | 0.0002368 | 0.003303 | -2.43 | 20.29 |
| 24 | transmembrane receptor protein serine/threonine kinase signaling pathway (GO:0007178) | 0.00006830 | 0.001567 | -2.09 | 20.06 |
| 25 | regulation of cell proliferation (GO:0042127) | 2.301e-8 | 0.000005067 | -1.13 | 19.85 |
| 26 | regulation of intracellular signal transduction (GO:1902531) | 0.00003233 | 0.0008980 | -1.91 | 19.79 |
| 27 | regulation of myosin-light-chain-phosphatase activity (GO:0035507) | 0.0002368 | 0.003303 | -2.36 | 19.70 |
| 28 | ventricular septum morphogenesis (GO:0060412) | 0.00003608 | 0.0009690 | -1.92 | 19.66 |
| 29 | small GTPase mediated signal transduction (GO:0007264) | 0.00001142 | 0.0004490 | -1.72 | 19.61 |
| 30 | ventricular septum development (GO:0003281) | 0.00008437 | 0.001717 | -2.07 | 19.42 |

**S8b Table.** Top 30 - GO Cellular Component 2018

| **Index** | **Name** | **P-value** | **Adjusted p-value** | **Z-score** | **Combined score** |
| --- | --- | --- | --- | --- | --- |
| 1 | MHC protein complex (GO:0042611) | 2.561e-7 | 0.00001895 | -2.11 | 32.05 |
| 2 | COPII-coated ER to Golgi transport vesicle (GO:0030134) | 0.000002958 | 0.00005471 | -2.10 | 26.73 |
| 3 | integral component of lumenal side of endoplasmic reticulum membrane (GO:0071556) | 0.000001767 | 0.00004359 | -1.94 | 25.71 |
| 4 | ER to Golgi transport vesicle membrane (GO:0012507) | 5.838e-7 | 0.00002160 | -1.76 | 25.26 |
| 5 | axon (GO:0030424) | 0.0008189 | 0.006733 | -1.99 | 14.12 |
| 6 | phagocytic vesicle membrane (GO:0030670) | 0.0002066 | 0.003058 | -1.56 | 13.22 |
| 7 | integral component of endoplasmic reticulum membrane (GO:0030176) | 0.0005714 | 0.005285 | -1.75 | 13.04 |
| 8 | spanning component of plasma membrane (GO:0044214) | 0.02047 | 0.07973 | -3.11 | 12.09 |
| 9 | death-inducing signaling complex (GO:0031264) | 0.02624 | 0.09248 | -3.19 | 11.62 |
| 10 | recycling endosome membrane (GO:0055038) | 0.0003838 | 0.004597 | -1.43 | 11.25 |
| 11 | dendrite (GO:0030425) | 0.003786 | 0.02155 | -1.93 | 10.76 |
| 12 | early endosome membrane (GO:0031901) | 0.001362 | 0.01008 | -1.54 | 10.13 |
| 13 | recycling endosome (GO:0055037) | 0.0004348 | 0.004597 | -1.24 | 9.56 |
| 14 | early endosome (GO:0005769) | 0.004240 | 0.02241 | -1.66 | 9.06 |
| 15 | late endosome lumen (GO:0031906) | 0.02912 | 0.09794 | -2.50 | 8.85 |
| 16 | phagocytic vesicle (GO:0045335) | 0.001961 | 0.01319 | -1.41 | 8.80 |
| 17 | spanning component of membrane (GO:0089717) | 0.03484 | 0.1121 | -2.24 | 7.51 |
| 18 | cytoplasmic vesicle (GO:0031410) | 0.003786 | 0.02155 | -1.26 | 7.03 |
| 19 | MHC class II protein complex (GO:0042613) | 0.04336 | 0.1284 | -2.13 | 6.69 |
| 20 | integral component of plasma membrane (GO:0005887) | 0.009966 | 0.04609 | -1.45 | 6.69 |
| 21 | membrane raft (GO:0045121) | 0.005363 | 0.02646 | -1.28 | 6.68 |
| 22 | SCF ubiquitin ligase complex (GO:0019005) | 0.01149 | 0.05003 | -1.48 | 6.63 |
| 23 | caveola (GO:0005901) | 0.01231 | 0.05059 | -1.33 | 5.86 |
| 24 | perinuclear region of cytoplasm (GO:0048471) | 0.02552 | 0.09248 | -1.53 | 5.62 |
| 25 | serine/threonine protein kinase complex (GO:1902554) | 0.07398 | 0.1781 | -2.07 | 5.40 |
| 26 | MLL1 complex (GO:0071339) | 0.07944 | 0.1781 | -2.08 | 5.27 |
| 27 | endosome lumen (GO:0031904) | 0.07124 | 0.1781 | -1.94 | 5.13 |
| 28 | MLL1/2 complex (GO:0044665) | 0.07944 | 0.1781 | -1.97 | 4.98 |
| 29 | Golgi membrane (GO:0000139) | 0.04162 | 0.1283 | -1.47 | 4.67 |
| 30 | contractile fiber (GO:0043292) | 0.07672 | 0.1781 | -1.80 | 4.61 |

**S8c Table.** Top 30-GO Molecular Function 2018

| **Index** | **Name** | **P-value** | **Adjusted p-value** | **Z-score** | **Combined score** |
| --- | --- | --- | --- | --- | --- |
| 1 | 1-phosphatidylinositol-3-kinase regulator activity (GO:0046935) | 0.0001780 | 0.002986 | -3.33 | 28.79 |
| 2 | vascular endothelial growth factor-activated receptor activity (GO:0005021) | 0.0002368 | 0.003417 | -3.12 | 26.08 |
| 3 | phosphatidylinositol 3-kinase regulator activity (GO:0035014) | 0.0003039 | 0.003417 | -2.98 | 24.11 |
| 4 | type I transforming growth factor beta receptor binding (GO:0034713) | 0.0003039 | 0.003417 | -2.82 | 22.81 |
| 5 | protein kinase C activity (GO:0004697) | 0.0004626 | 0.004657 | -2.87 | 22.05 |
| 6 | protein serine/threonine kinase activity (GO:0004674) | 1.138e-7 | 0.00001719 | -1.26 | 20.18 |
| 7 | growth factor activity (GO:0008083) | 0.000001964 | 0.0001176 | -1.47 | 19.31 |
| 8 | neurotrophin TRK receptor binding (GO:0005167) | 0.0003039 | 0.003417 | -2.34 | 18.95 |
| 9 | transmembrane receptor protein serine/threonine kinase activity (GO:0004675) | 0.0008764 | 0.007352 | -2.45 | 17.28 |
| 10 | kinase binding (GO:0019900) | 0.00003022 | 0.0009127 | -1.57 | 16.31 |
| 11 | cyclin-dependent protein kinase activity (GO:0097472) | 0.0001492 | 0.002817 | -1.83 | 16.10 |
| 12 | cyclin-dependent protein serine/threonine kinase activity (GO:0004693) | 0.0001367 | 0.002817 | -1.79 | 15.90 |
| 13 | protein kinase activator activity (GO:0030295) | 0.00002237 | 0.0008445 | -1.44 | 15.45 |
| 14 | protein serine/threonine kinase inhibitor activity (GO:0030291) | 0.003765 | 0.02187 | -2.60 | 14.53 |
| 15 | protein kinase activity (GO:0004672) | 0.000002337 | 0.0001176 | -1.09 | 14.12 |
| 16 | transforming growth factor beta receptor binding (GO:0005160) | 0.0003168 | 0.003417 | -1.74 | 14.05 |
| 17 | BMP receptor activity (GO:0098821) | 0.02047 | 0.07025 | -3.48 | 13.53 |
| 18 | MAP kinase kinase kinase activity (GO:0004709) | 0.001645 | 0.01080 | -2.07 | 13.29 |
| 19 | interleukin-6 receptor binding (GO:0005138) | 0.02336 | 0.07349 | -3.33 | 12.50 |
| 20 | transmembrane receptor protein tyrosine kinase activity (GO:0004714) | 0.0008150 | 0.007352 | -1.60 | 11.39 |
| 21 | protein homodimerization activity (GO:0042803) | 0.003314 | 0.02002 | -1.99 | 11.39 |
| 22 | activin-activated receptor activity (GO:0017002) | 0.02336 | 0.07349 | -3.01 | 11.31 |
| 23 | transforming growth factor beta-activated receptor activity (GO:0005024) | 0.02047 | 0.07025 | -2.87 | 11.16 |
| 24 | growth factor receptor binding (GO:0070851) | 0.002621 | 0.01649 | -1.88 | 11.16 |
| 25 | Arp2/3 complex binding (GO:0071933) | 0.02336 | 0.07349 | -2.87 | 10.77 |
| 26 | kinase activity (GO:0016301) | 0.001424 | 0.009773 | -1.60 | 10.48 |
| 27 | inositol tetrakisphosphate phosphatase activity (GO:0052743) | 0.02912 | 0.08296 | -2.94 | 10.38 |
| 28 | protein kinase binding (GO:0019901) | 0.00009877 | 0.002486 | -1.09 | 10.08 |
| 29 | protein serine/threonine kinase activator activity (GO:0043539) | 0.004009 | 0.02242 | -1.81 | 9.97 |
| 30 | neurotrophin TRKA receptor binding (GO:0005168) | 0.02047 | 0.07025 | -2.56 | 9.94 |
