# Supplementary figures and images for: Induction of miR 21 impairs the anti-Leishmania response through inhibition of IL-12 in canine splenic leukocytes
Source: PLoS One. 2019 Dec 11;14(12):e0226192. doi: 10.1371/journal.pone.0226192 (PMC6905561; doi:10.1371/journal.pone.0226192)

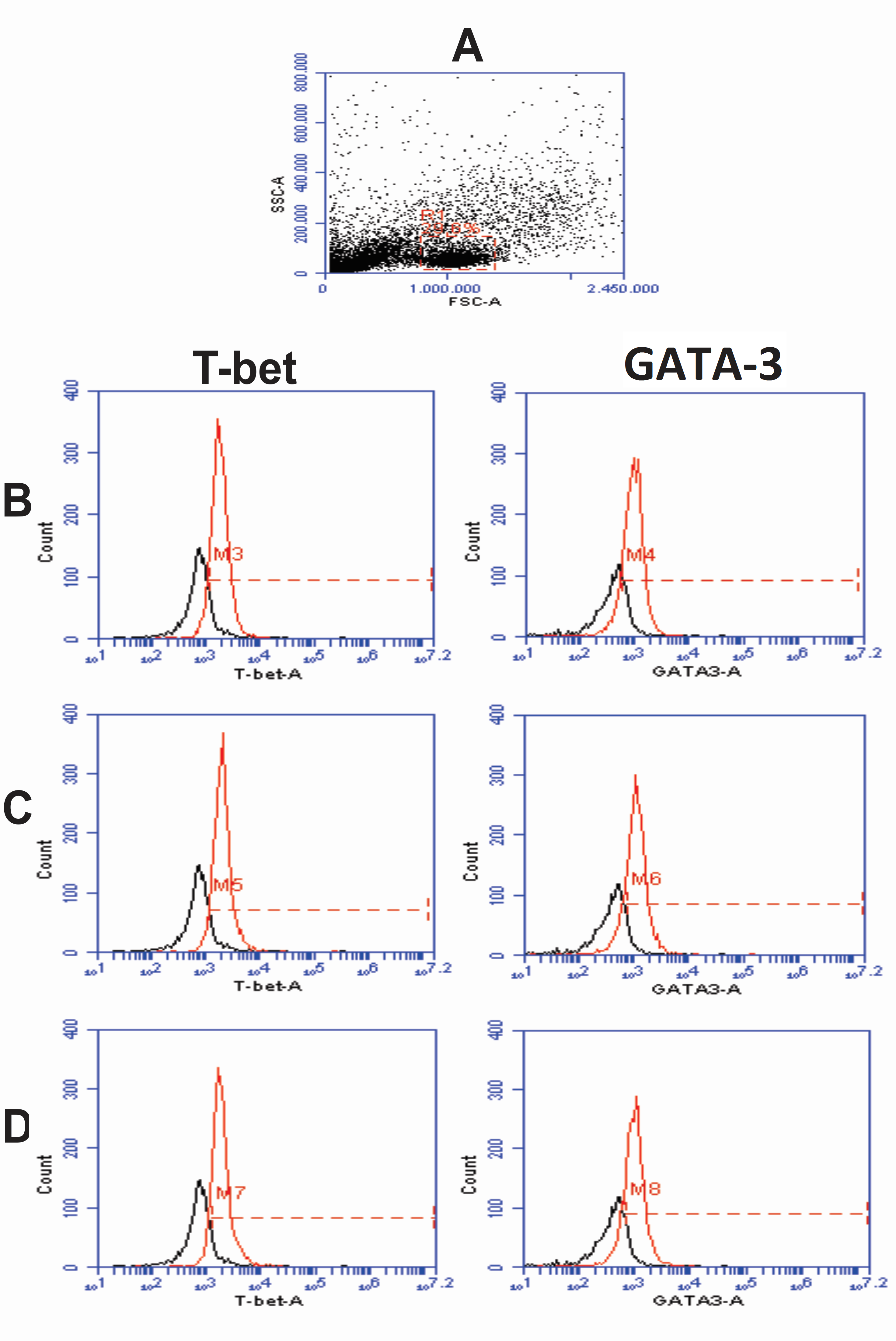

Supplement: S1 Fig — Selected lymphocyte population (A), in the presence of a miR 21 mimic (B), in the presence of a Negative control (scrambled) (C), in the presence of a miR 21 Inhibitor (D). Gate in R is a lymphoid cell mark, gate in M marks T-bet and GATA-3, red peak marks T-bet and GATA-3 positive cells and black peak is positive for their respective isotypes control. (TIF) [file pone.0226192.s009.tif]

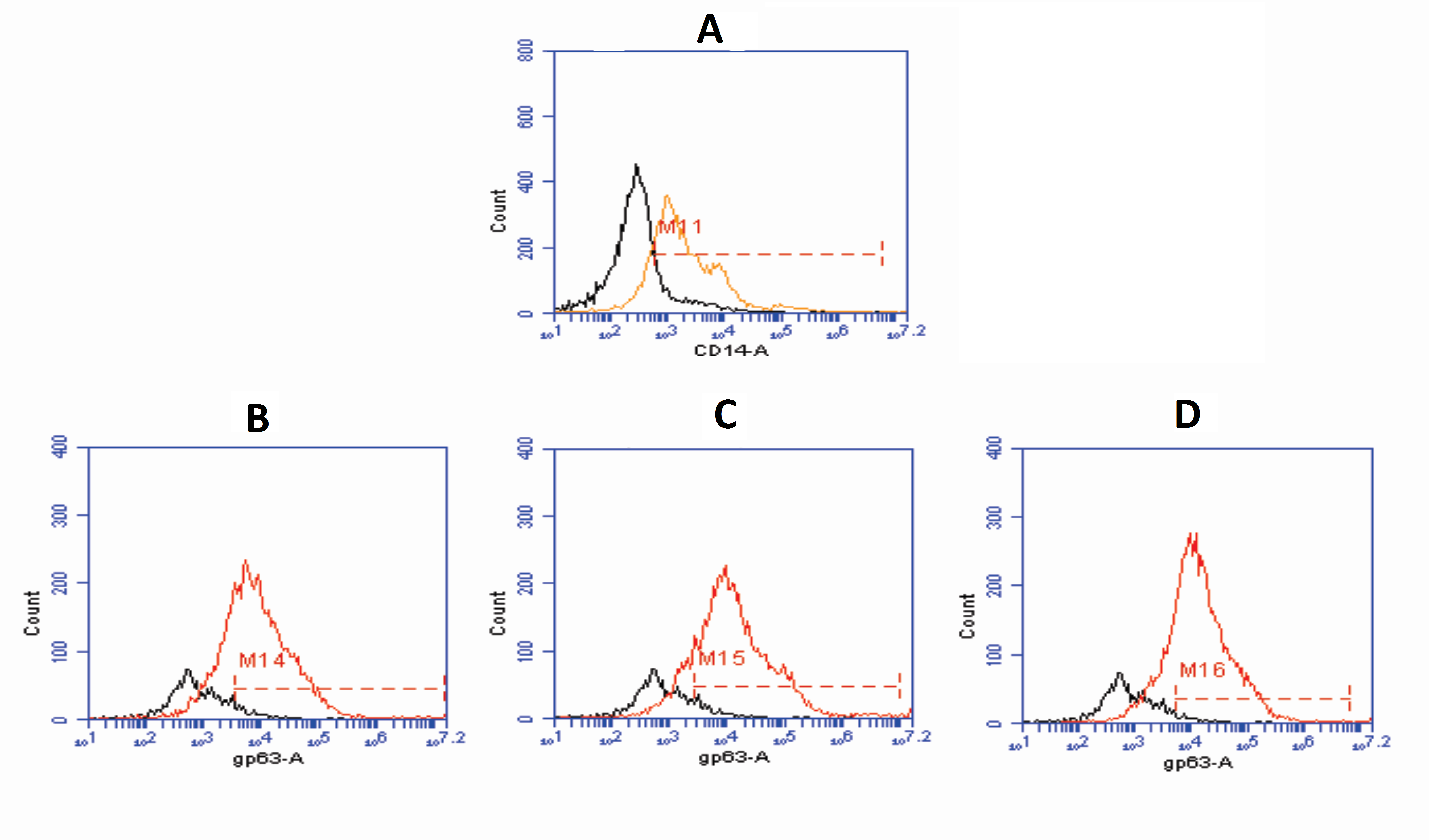

Supplement: S2 Fig — (A) Orange peak population labeled with CD14+ (M11), red peak positivity for gp63 and CD14+ cell (B) in the presence of a miR 21 Mimic (C) in the presence of a negative control (scrambled) (D) and in the presence of the Inhibitor of miR 21 (D). (TIF) [file pone.0226192.s010.tif]
